# Effects of photobiomodulation on interleukin-10 and nitrites in individuals with relapsing-remitting multiple sclerosis – Randomized clinical trial

**DOI:** 10.1371/journal.pone.0230551

**Published:** 2020-04-07

**Authors:** Tamiris Silva, Yara Dadalti Fragoso, Maria Fernanda Setúbal Destro Rodrigues, Andréa Oliver Gomes, Fernanda Cordeiro da Silva, Lucas Andreo, Ariane Viana, Daniela de Fátima Teixeira da Silva, Maria Cristina Chavantes, Anna Carolina Ratto Tempestini Horliana, Kátia De Angelis, Alessandro Melo Deana, Luciana Prats Branco, Kristianne Porta Santos Fernandes, Lara Jansiski Motta, Raquel Agnelli Mesquita-Ferrari, Sandra Kalil Bussadori

**Affiliations:** 1 Universidade Nove de Julho, UNINOVE, São Paulo, SP, Brazil; 2 Universidade Metropolitana de Santos, UNIMES, Santos, SP, Brasil; 3 Escola Paulista de Medicina, Universidade Federal de São Paulo (UNIFESP), São Paulo, SP, Brasil; University Medical Center Gottingen, GERMANY

## Abstract

**Objective:**

Investigate the effects of photobiomodulation (PBM) on the expression of IL-10 and nitrites in individuals with Relapsing-Remitting multiple sclerosis (MS), as these biomarkers play a fundamental role in the physiopathology of the disease. The modulation of IL-10 and nitrites through treatment with PBM may be a novel treatment modality for MS.

**Methods:**

A randomized, uncontrolled, clinical trial was conducted involving 14 individuals with a diagnosis of Relapsing-Remitting MS and a score of up to 6.0 on the Expanded Disability Status Scale (EDSS).

**The participants were randomized to two groups:**

Group 1 –PBM in the sublingual region; Group 2 –PBM over the radial artery. Irradiation was administered with a wavelength of 808 nm and output power of 100 mW for 360 seconds twice a week, totaling 24 sessions. Peripheral blood was analyzed for the determination of serum levels of IL-10 and nitrites.

**Results:**

After treatment with PBM, the expression of IL-10 increased in both the sublingual group (pre-treatment: 2.8 ± 1.4 pg/ml; post-treatment: 8.3 ± 2.4 pg/ml) and the radial artery group (pre-treatment: 2.7 pg/ml ± 1.4; post-treatment: 11.7 ± 3.8 pg/ml). In contrast, nitrite levels were not modulated in the sublingual group (pre-treatment: 65 ± 50 nmol/mg protein; post-treatment: 51 ± 42 nmol/mg protein) or the radial artery group (pre-treatment: 51 ± 16 nmol/mg protein; post-treatment: 42 ± 7 nmol/mg protein).

**Conclusion:**

Treatment with PBM positively modulated the expression of IL-10 but had no effect on nitrite levels. Further studies should be conducted with a larger sample and a control group, as PBM may be a promising complementary treatment for the management of MS.

This trial is registered at ClinicalTrials.gov. Identifier: NCT03360487.

## Introduction

The central nervous system (CNS) can be affected by numerous adverse conditions, such as multiple sclerosis (MS), which is an autoimmune disease classified as the second most common cause of physical disability, especially among young individuals.[1,2] One of the prominent characteristics of MS is the infiltration of Th1 lymphocytes from peripheral blood into the brain and spinal cord, leading to the activation of microglia.[3]

Microglia play an important role in MS and are capable of differentiating into two phenotypes: M1 (pro-inflammatory) and M2 (anti-inflammatory).[4] The M1 phenotype is activated when Th1 lymphocytes invade the CNS. Thus, microaglia act as neurotoxic cells, which not only produce pro-inflammatory cytokines [2], but also release high concentrations of nitric oxide and its metabolites, such as nitrites, contributing to oxidative stress and demyelination.[2,5]

Demyelination results in a reduction in nerve impulses, causing multiple symptoms, such as visual disorders, spasticity, muscle weakness, compromised gait, coordination difficulties, tremors/ataxia, neuropathic pain, cognitive and sensory deficits, emotional changes and fatigue.[6] The measurement of functional disability is fundamental to the monitoring of the course of the disease and the effect of treatment. The Expanded Disability Status Scale (EDSS) is the gold standard for quantifying disability in individuals with MS, the score of which ranges from 0 to 10 points.[7,8]

The M2 phenotype of microglia is induced by anti-inflammatory cytokines, such as interleukin (IL)-10, which leads to the occurrence of tissue repair. This cytokine plays important roles in the human immune response. It is an important inhibitor of Th1 cytokines and deactivator of monocytes/macrophages, blocking the synthesis of pro-inflammatory cytokines. Since this is an important mechanism for the treatment of MS. [9,10]

Currently, there is no cure for MS, although there are medications that can delay the progression of the disease.[10,11] Thus, strategies for increasing IL-10 may be effective, as individuals with MS have lower levels of IL-10 compared to healthy individuals.[12] Photobiomodulation (PBM) is a low-level light source capable of stimulating or inhibiting cellular processes.[13] Evidence suggests that light is absorbed by chromophores, leading to greater cellular respiration, the formation of ATP as well as the modulation of oxidative/nitrosative stress and IL-10.[6,14] Thus, PBM may be a promising strategy to complement the treatment of MS.

The aim of the present study was to investigate the effects of PBM on the expression of IL-10 and nitrites in individuals with Relapsing-Remitting MS, as these biomarkers play an important role in the physiopathology of the disease. The results of this study are expected to reveal a novel treatment modality for MS.

## Methods

A randomized, uncontrolled, blind, clinical trial was developed at the Integrated Health Clinic of University Nove de Julho (UNINOVE). This study was conducted in accordance with the norms governing research involving human subjects (Resolution 466/2012) and received approval from the UNINOVE Human Research Ethics Committee (certificate number: 2.423.755). The participants or their legal guardians authorized participation in the study by signing a statement of informed consent (S2 File). The individual pictured in Fig 2 has provided written informed consent (as outlined in PLOS consent form) to publish their image alongside the manuscript. The Consolidated Standards for Reporting Trials (CONSORT statement) were followed to ensure quality and transparency (S5 File). The protocol for this study was registered with ClinicalTrials (ClinicalTrials.gov Identifier: NCT03360487).

### Study design

The participants were recruited from the UNINOVE integrated health clinic based on the following eligibility criteria:

Inclusion criteria: Men and women aged 18 to 60 years with Relapsing-Remitting MS under pharmacological treatment, capable of understanding and responding to verbal stimuli, with an EDSS score of up to 6 points.

Exclusion criteria: Other autoimmune diseases, heart, respiratory, kidney or liver failure, acquired immunodeficiency syndrome and pregnant women.

The collection period was from May (2018) to October (2018). Screening was performed by telephone contact with patients in treatment and on the waiting list at the UNINOVE clinic. Patients who met the eligibility criteria underwent a physiotherapeutic and medical evaluation, during which the baseline EDSS was determined. After the collection of blood, the participants were randomized and allocated to the different groups.

### Participants

**Fig 1:**
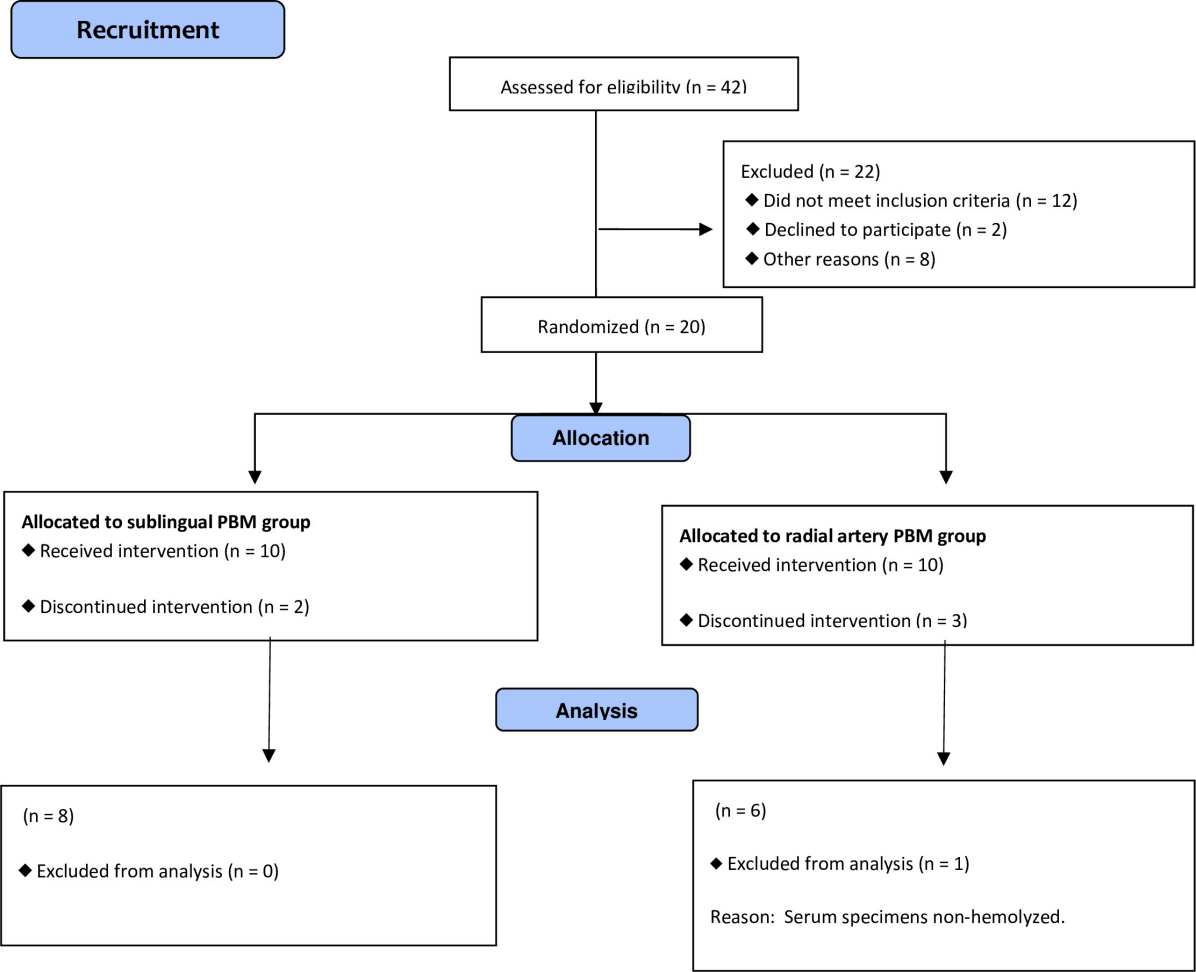
Flowchart CONSORT 2010.

Forty-two individuals were screened and 22 were excluded: 12 did not meet the inclusion criteria, two declined to participate in the study and eight were unable to participate due to the fact that the study schedule conflicted with their work schedule. Thus, 20 individuals were randomized to two groups. Ten were allocated to the sublingual PBM group (Group 1) but two dropped out during the course of the intervention. Ten individuals were allocated to the radial artery PBM group (Group 2) but three dropped out during the course of the intervention, totaling seven individuals in this group. However, one participant in Group 2 was excluded from the analysis due to non-hemolyzed serum samples (Fig 1).

Thus, the final sample was composed of 15 individuals (Table 1). All participants in both groups continued taking their routine medications for the base condition. No placebo control group was used due to the difficulty in recruiting participants. Thus, only the two active PBM groups were used.

**Table 1:** Characteristics of participants.

Participant	Group	Age	Sex	EDSS	Medication
A	1	47	F	4	Beta interferon 1 A
B	1	42	F	2	Glatiramer acetate
C	1	59	F	6	Fingolimod
D	1	37	F	5	Beta interferon 1 A
E	1	41	M	2	Fingolimod
F	1	31	F	1	Beta interferon 1A
G	1	37	F	1	Natalizumab
H	1	30	F	4.5	Dimethyl fumarate
I	2	23	F	2.5	Glatiramer acetate
J	2	37	F	3	Dimethyl fumarate
L	2	45	F	3	Beta interferon 1 A
M	2	24	F	2	Dimethyl fumarate
N	2	47	F	4	Beta interferon
O	2	37	F	3	Dimethyl fumarate
P*	2	37	M	2	Interferon 1 B

F = female; M = male; Medications–first line: beta interferon 1A, interferon 1B, glatiramer acetate; second line: fingolimod, natalizumab, dimethyl fumarate.

* Individual excluded from analysis

### Photobiomodulation

**Fig 2:**
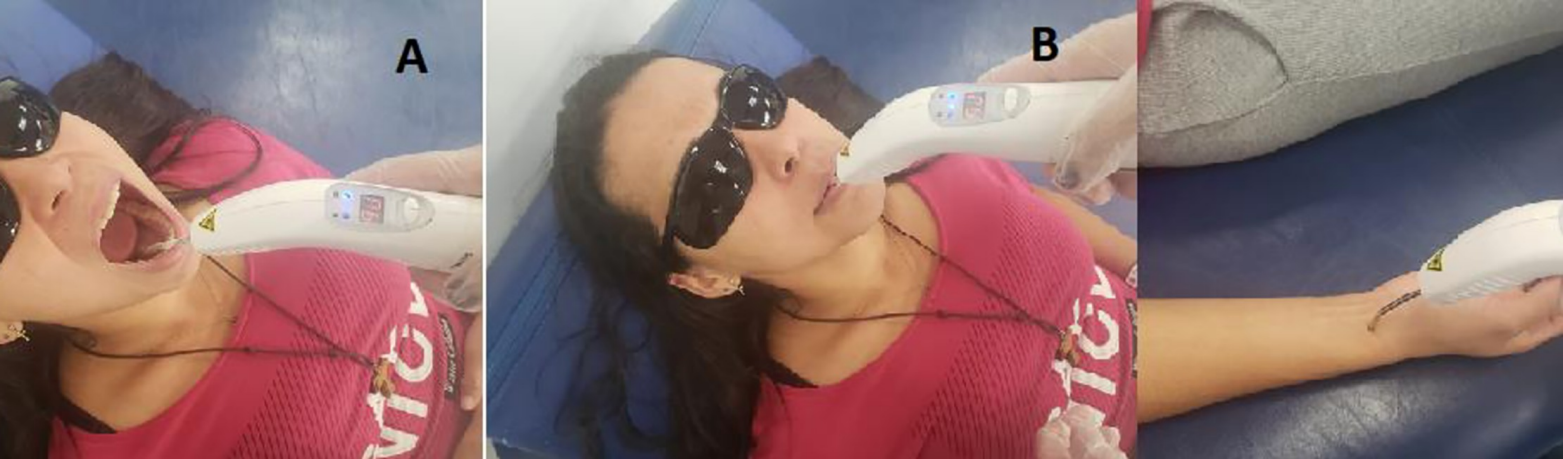
Positioning for irradiation. A. Opening of mouth for positioning of laser tip in sublingual region. B. Patient with closed mouth to enable irradiation of sublingual region. C. After palpation of radial artery, laser tip positioned for irradiation.

For irradiation, the individuals were placed either in the sitting position with the head supported or placed comfortably in the supine position with the upper limb positioned on an examining table (Fig 2).

For sublingual administration, plastic wrap was placed on the application pen. The patient was instructed to open his/her mouth and lift the tongue to enable the positioning of the laser pen. For transcutaneous administration over the radial artery, the operator performed palpation to determine the positioning of the tip of the laser pen. After setting the parameters based on the literature, the PBM treatment protocol was established. The same PBM parameters were used in both groups (wavelength: 808 nm; power output: 100 mW; diameter: 0.40 cm; area: 0.13 cm^2^; irradiance: 0.80 W/cm^2^; radiant exposure: 287.00 J/cm^2^; energy: 36.05 J; exposure time: 360 seconds. Sessions were held twice a week for 12 weeks (total: 24 sessions). The same operator performed the irradiations in all sessions.

### Physical therapy protocol

**Fig 3:**
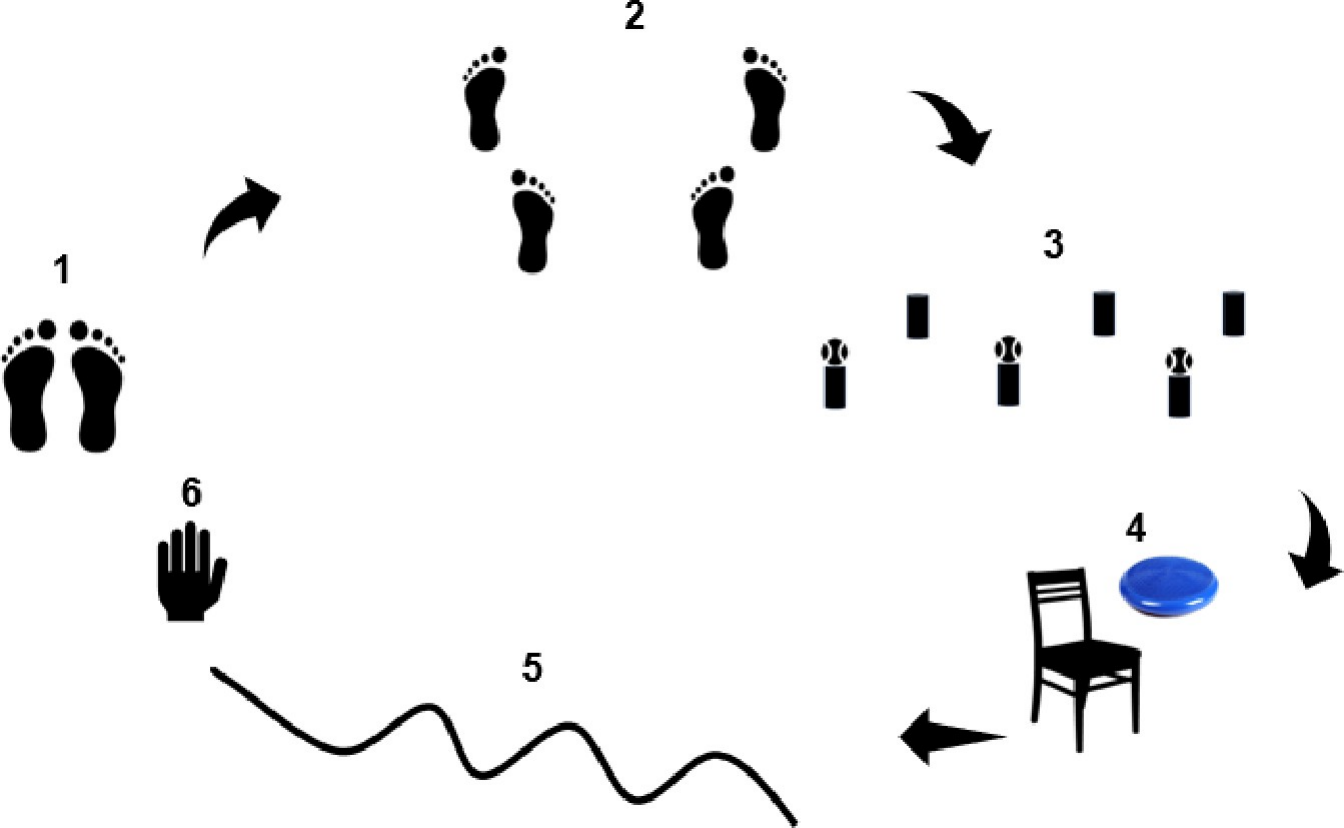
Physical therapy. 1) Feet together for 30 seconds with eyes open and 30 seconds with eyes closed on carpets with different textures; 2) Tandem position for 30 seconds with eyes closed and 30 seconds performing a dual task; 3) Squatting to pick up a ball from a cone, standing up, taking a step and placing the ball on another cone (three repetitions); 4) Sitting on chair with balancing disk and performing dual task of picking up objects from the sides (three times on each side); 5) Walking three meters while answering questions posed by therapist or transporting objects; 6) Two minutes of rest.

Physical therapy was performed twice a week after PBM and involved exercises at several stations of a circuit: Feet together; feet tandem on a variety of different surfaces (hard, foam rubber and carpets with different textures) and different sensory inputs (eyes open or closed); walking forward and backward on firm or foam rubber surfaces; walking through obstacles; and squatting. At each station, a cognitive task was included, such as naming objects, and the participants were instructed to transport light objects from one place to another (Fig 3).[15,16]

### Blood collection

Although the immune response in MS is located mainly in the CNS, abnormalities have also been found in the peripheral blood [3]. Blood collection was performed by a blinded examiner. Ten ml of blood were collected from the median cubital vein of the participants. Centrifugation was performed for 10 minutes at a velocity of 2,800 rotations per minute. The serum layer was removed at aliquots of 300 microliters (μL) per 1.5-mL tube for each patient and stored at -80°C until use.

### Evaluations

#### Protein expression of IL-10

Cytokine IL-10 was analyzed by a blinded assessor and quantified from serum samples of the individuals using ELISA MAX HUMAN kits (BioLegend), which contain capture and detection antibodies for the precise quantification of each cytokine. All kits were used following the manufacturer's instructions. The optical density of the samples was measured in a spectrophotometer at 450 nm. http://dx.doi.org/10.17504/protocols.io.92dh8a6.

#### Nitrite levels

The quantification of nitrites in the serum samples of the individuals was determined using the Griess test. The samples were incubated with the Griess reagent at room temperature for 10 minutes and optical density was measured with a spectrophotometer at 492 nm. Blind assessment http://dx.doi.org/10.17504/protocols.io.96ph9dn

#### Changes in relation to the original protocol

It was necessary to make changes to the previously published study protocol [17]. No group received irradiation along the spinal column due to the difficulty in palpating the vertebrae to locate the irradiation point in obese individuals. Due to the small number of patients at the Integrated Health Clinic at the time of recruitment and the lack of an appropriate device for simulating treatment, the leader of the study decided to concentrate on the effect sizes and the comparison of irradiation sites rather than having an unblinded control group. Thus, the decision was made to randomize the patients into two treatment groups.

The evaluation of TNF-α could not be performed because the material for analysis was not available at the time of the study.

#### Statistical analysis

The data were tabulated and treated using Graph Pad Prism version 7.0. The Shapiro-Wilk test was used to assess the distribution of the data and the p-values were rectified using the Ryan-Holm stepdown Bonferroni procedure for the multiple tests. Data with Gaussian distribution were expressed as mean and standard deviation values and non-parametrical data were expressed as median and the range (min, max) was calculated. Comparisons between groups were performed using two-way repeated-measures ANOVA for parametric data and the Mann-Whitney test for non-parametric data. Pair-wise comparisons were performed using paired t-test. Correlations between variables were tested using Spearman’s test. The significance level was set at α = 0.05.

## Results

Fifteen individuals participated in the study, thirteen women and two men. No significant differences between groups were found regarding age or EDSS score (median [range]: 3.0 [1.00; 6.0] in the sublingual group and 3.0 [2.00; 4.00] in the radial artery group) (Mann-Whitney test)

**Fig 4:**
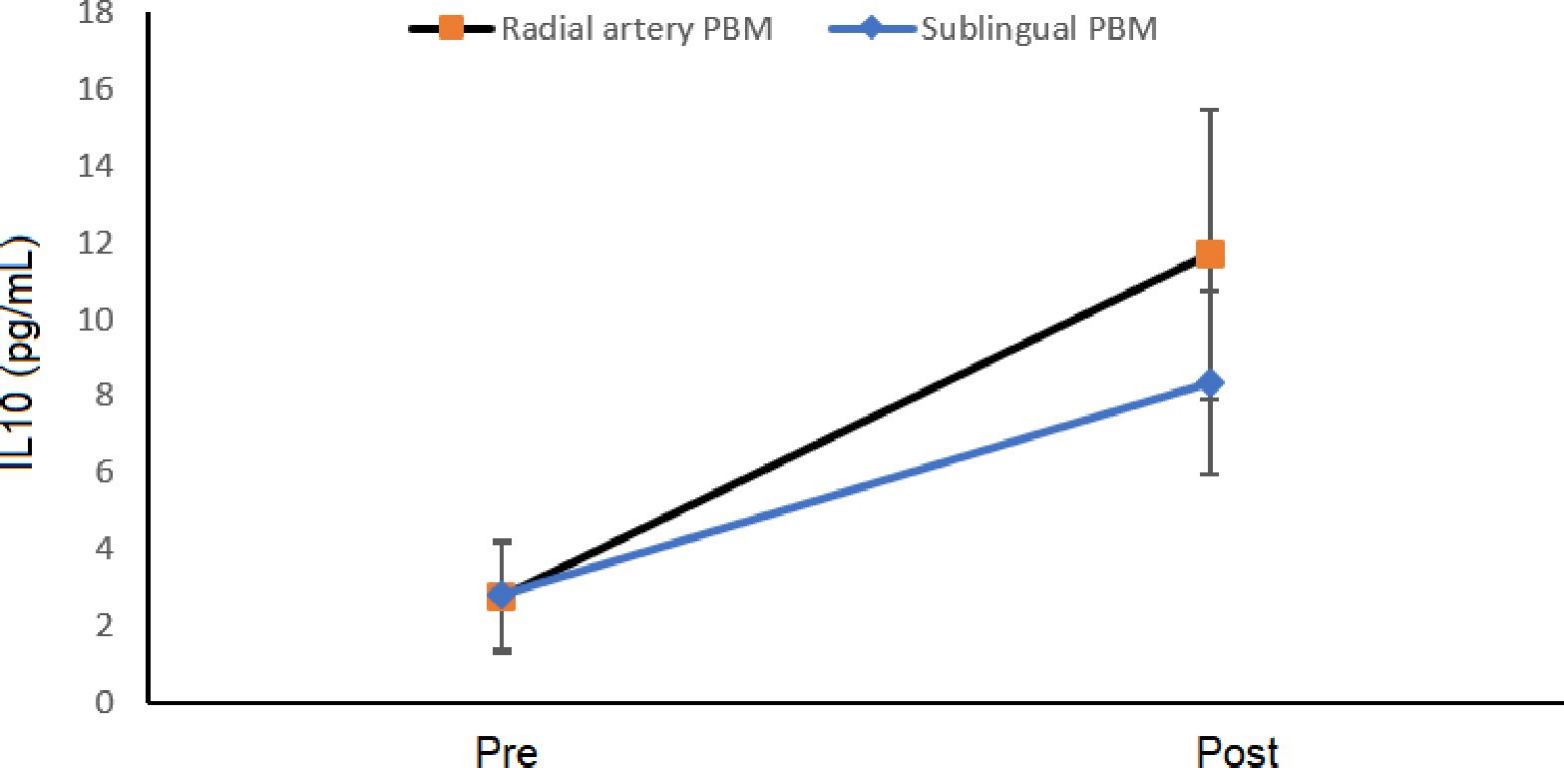
Expression of interleukin-10. Values in both groups before (pre) and after (post) treatment with PBM (p = 0.008, two-way ANOVA).

After treatment with PBM, the expression of IL-10 increased significantly in both the sublingual group (pre-treatment: 2.8 ± 1.4 pg/ml; post-treatment: 8.3 ± 2.4 pg/ml, p = 0.015, paired t-test) and radial artery group (pre-treatment: 2.7 ± 1.4 pg/ml; post-treatment: 11.7 ± 3.8 pg/ml, p = 0.031, paired t-test) (Fig 4). Moreover, no significant difference was found between irradiation sites (p = 0.513, two-way repeated-measures ANOVA) and no interaction was found (p = 0.494, two-way repeated-measures ANOVA).

**Fig 5:**
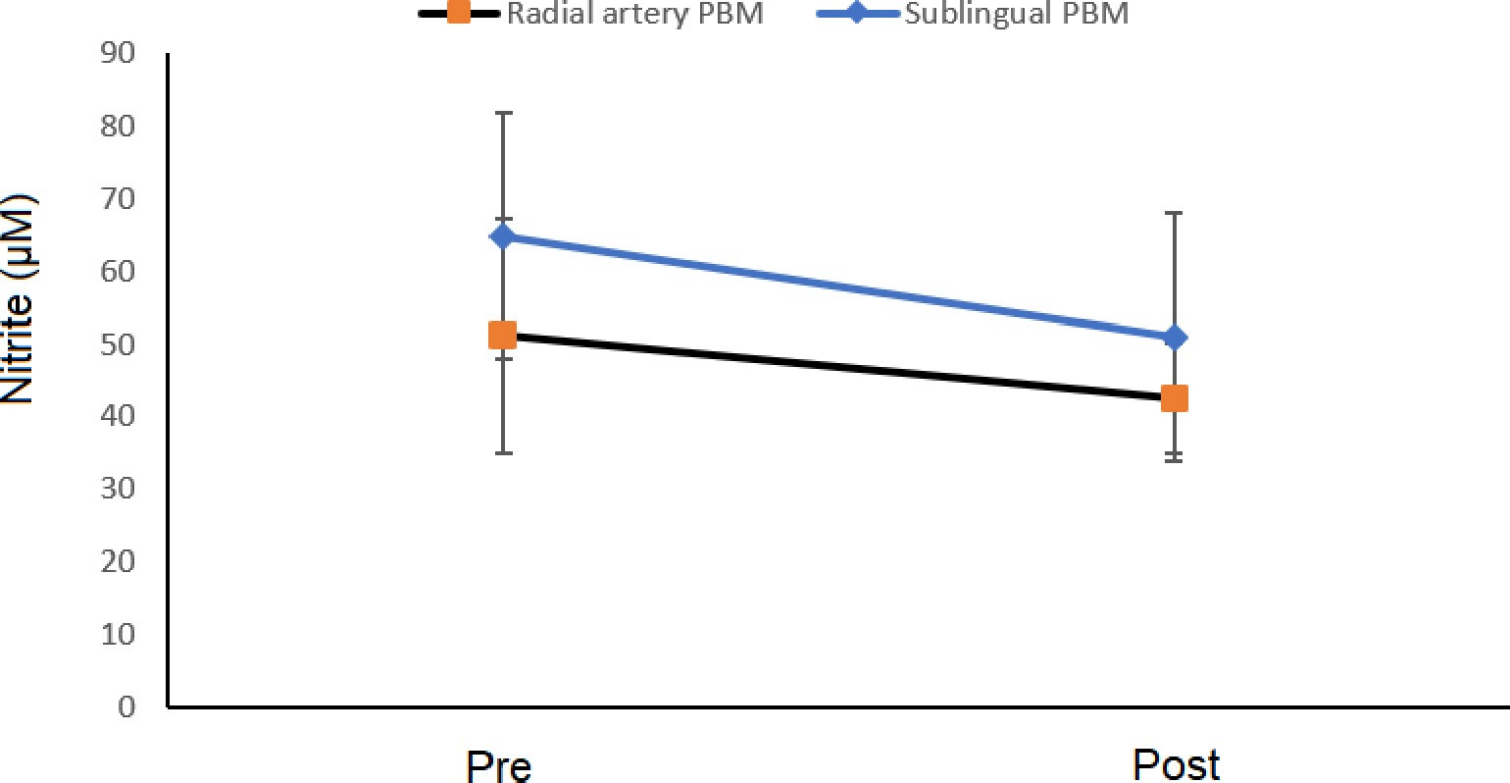
Nitrite levels. Values in both groups before (pre) and after (post) treatment with PBM (p = 0.543, two-way repeated-measures ANOVA).

**Fig 6:**
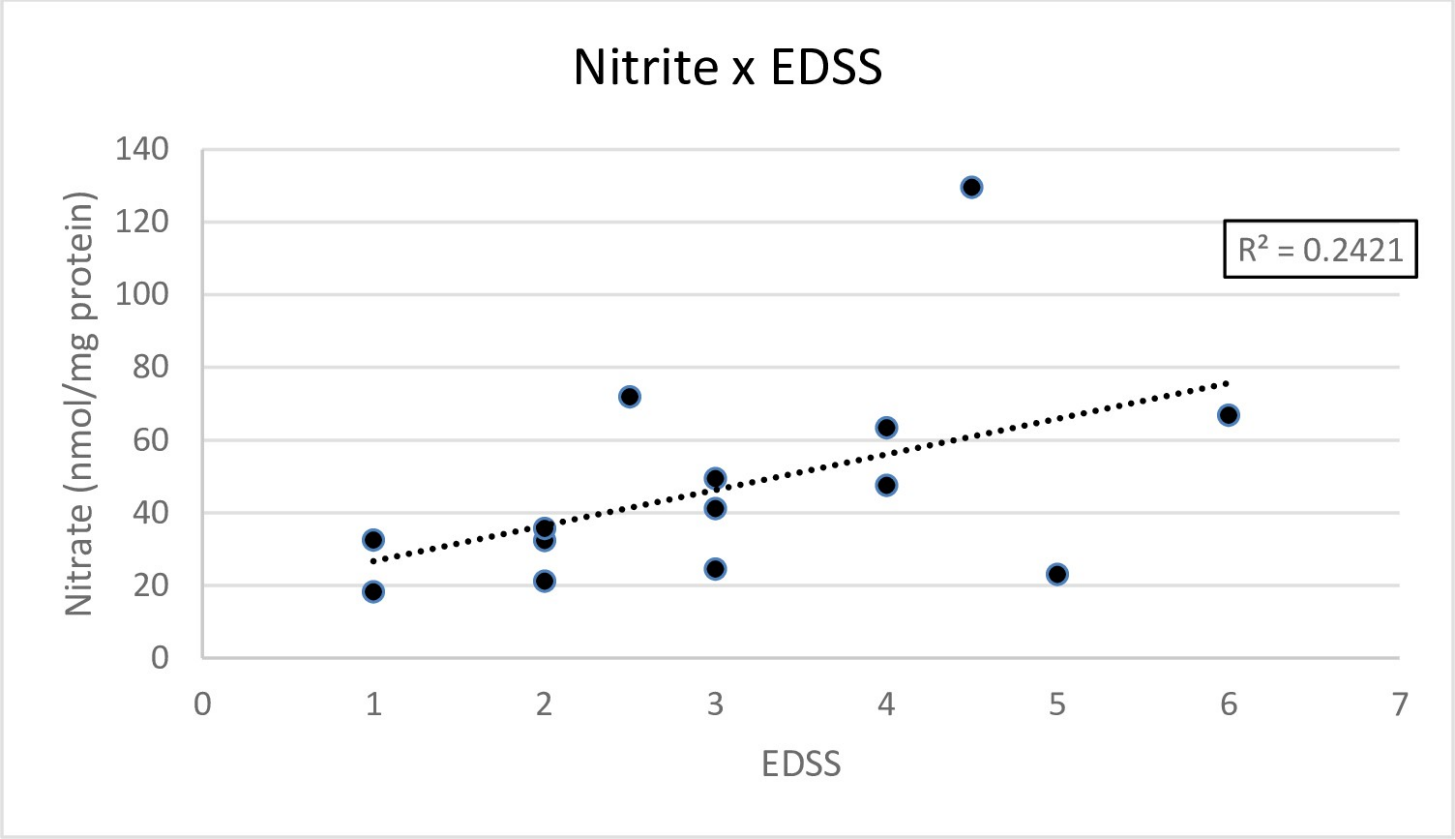
Nitrite levels. Correlation between EDSS score and nitrite levels. (r = 0.52, p = 0,0542).

In contrast, nitrite levels did not diminish significantly in either the sublingual group (pre-treatment: 65 ± 50 nmol/mg protein; post-treatment: 51 ± 42 nmol/mg protein) or radial artery group (pre-treatment: 51 ± 16 nmol/mg protein; post-treatment: 42 ± 7 nmol/mg protein) (Fig 5). No significant difference was found between irradiation sites (p = 0.5428, two-way repeated-measures ANOVA), no interaction was found (p = 0.8596, two-way repeated-measures ANOVA) and no significant difference was found between the periods analyzed (p = 0.5631, two-way repeated-measures ANOVA). An analysis involving all MS patients (sublingual and radial artery groups) demonstrated a positive correlation between EDSS score and nitrite levels post treatment (r = 0.52, p = 0,0542). (Fig 6).

## Discussion

Individuals with MS are treated by a multidisciplinary team in an individual manner.[18] However, medical and physiotherapeutic treatments are limited and new therapeutic modalities are needed to impede the progression of the disease.[10] The present study demonstrated that both forms of irradiation were effective at increasing the expression of IL-10 and may be used to complement the management of MS.

Serum nitrites are considered the main end products of the endogenous synthesis of nitric oxide. At high concentrations, nitric oxide stimulates the inflammatory response, increases the permeability of the blood-brain barrier, induces mitochondrial dysfunction, diminishes the energy metabolism of neural cells and consequently accelerates the death of oligodendrocytes.[19] In a cross-sectional study, Giovannoni et al [20] found that serum nitrate and nitrite levels are high in patients with MS.

According to Tang et al,[19] oxidative stress in MS causes injury to axons, mitochondrial dysfunction and dysfunction of the energy metabolism, especially in oligodendrocytes, accelerating the death of these cells. Moreover, nitric oxide inhibits the expression of genes related to the formation of myelin. All these findings suggest that oxidative stress plays a crucial role in neurodegeneration, which can lead to permanent sequelae and greater disability. The present results are in agreement with this suggestion, as we found a positive correlation between the EDSS scores and systemic levels of nitrite, indicating that individuals with MS with a greater degree of disability exhibit higher nitrite levels. This finding offers further evidence of role of nitrosative stress in dysfunctions associated with MS.

Reductions in nitrite levels have been found following *in vitro* e *in vivo* PBM in studies by Muili et al [14,21], Von Leden et al(4) and Song et al [22]. The authors also found that treatment offered neuroprotection and led to a clinical improvement in the experimental autoimmune encephalomyelitis model. Likewise, Wong-Riley et al [23] showed positive results of PBM in terms of reducing oxidative stress in neurons after treatment at an infrared wavelength, reporting an increase in energy metabolism that impeded neural death. In the present study, however, we found no significant reduction in nitrite levels, regardless of the form of irradiation.

However, Muili et al.[14] demonstrated in an experimental model of MS that cells treated with light produced less nitrites than the sham group throughout the course of the experiment. The cells were irradiated once a day for a period of 96 hours, and the reduction in nitrites was observed after 72 hours. In another study by Muili, et al,[21] the animals were irradiated and evaluated in 3 experimental periods of 48, 72 and 96 hours. They observed an increase in the expression of IL-10, in all experimental periods, in the PBM group. In both works, the authors used the same dosimetric parameters, but it took 72 hours to see a reduction in nitrites. This in comparison to the IL-10 in which modulation was seen after just 48 hours of irradiation. In fact, nitrites and IL-10 act in different ways. Nitric oxide and its metabolites, such as nitrites, play several roles in the progression of MS; they are related to axonal loss and disease progression, due to their pro-inflammatory characteristics. IL-10 has an anti-inflammatory characteristic. In our study, individuals were irradiated for only 360 seconds, twice a week for twenty-four sessions. One would expect, according to the work of Muili, et al, that a longer irradiation time would be necessary in order to see a significant reduction in nitrite, although there was an increase in IL-10 with just 360 seconds exposure. In future works aiming to evaluate the modulation of nitrites in MS it will be necessary to determine the minimum exposure time necessary to yield such an effect, since the parameters used in this study did not.

According to Kwilasz et al [12], IL-10 levels are lower in mononuclear cells of the peripheral blood in individuals with MS compared to healthy controls. It is noteworthy that the secretion of IL-10 by these cells is reduced prior to a relapse and increases during remission, suggesting that IL-10 is necessary for the occurrence of recovery. Molina et al [24] report similar data and state that IL-10 inhibits the expression of pro-inflammatory cytokines, neutralizing the inflammatory process. Thus, strategies designed to increase IL-10 may be effective in the treatment of autoimmune diseases, such as MS. Von Lenden et al [4] found high levels of IL-10 after the irradiation of microglia with a wavelength of 808 nm, which induced the M2 microglial phenotype, playing a significant role in the resolution of inflammation due to the expression of anti-inflammatory factors. Both forms of irradiation employed in the present study were designed to affect the blood. Similar results are described by Zhevago et al [7] who evaluated the effect of PBM on the blood stream and found that infrared light was capable of reducing proinflammatory cytokines and positively modulating the expression of anti-inflammatory cytokines. These findings are in agreement with the results of the present study, in which 24 sessions of PBM at a wavelength of 808 nm led to an increase in IL-10 levels in both treatment groups, independently of the medication the patients were taking.

According to Berkowitz et al. [25], there is a broad variety of protocols for investigating the response of cytokines to exercise. Exercise intensity is known to be an important variable and exercises of moderate to high intensity are the most widely used in an attempt to modulate cytokines. Some authors have demonstrated that both aerobic exercise [26] and resistance training [27] can improve the quality of life of these patients but were not found to exert an effect on anti-inflammatory cytokines.

Reference values for IL-10 in individuals with MS are scarce. However, the present data are compatible with findings described by Berkowitz et al. [25], who proposed an intervention based on moderate physical exercises for individuals with MS and healthy controls and found no statistically significant difference in IL-10 levels after the intervention compared to baseline in either group, concluding that physical exercise under the conditions reported did not exert an influence on this interleukin.

In contrast, Alvarenga-Filho et al. [28] evaluated the impact of a 12-week physical activity program consisting of the combination of Pilates exercises and 30 minutes of aerobic exercise on cytokine production. The results showed an increase in the anti-inflammatory effects of serotonin, as evidenced by the high production of IL-10. This seems to demonstrate the need for high intensity exercises to achieve immunomodulation in these patients.

Although some studies report that the repetitive practice of intensive physical exercise can alter the expression of IL-10, there is no evidence to state that moderate to low intensity exercise would have a similar effect. In the present study, physiotherapeutic exercises were performed, which included static and dynamic balance training with sensory and cognitive stimuli. Therefore, the change in IL-10 in this study may rather be due to the immunomodulating effect of PBM.

Limitations of the study: The lack of a control group was due to the small number of patients at the Integrated Health Clinic at the time of recruitment. Moreover, there was no appropriate device for simulating treatment. Another limitation was the fact that we did not perform functional tests, as physiotherapeutic exercises can lead to functional improvements in individuals with MS. Moreover, adequate palpation for the localization of the radial artery prior to irradiation depends on the skill of the operator, which may be a source of bias.

In conclusion, treatment with PBM positively modulated the expression of IL-10 but had no effect on nitrite levels. Further studies should be conducted with a larger sample and a control group, as PBM may be a promising complementary treatment for the management of MS.

## Supporting information

S1 File Original Ethics Committee in Research (Portuguese). (PDF)

S2 File Ethics Committee in Research (English). (PDF)

S3 File Declaration of consent for participation in clinical research original (Portuguese). (PDF)

S4 File Declaration of consent for participation in clinical research (English). (PDF)

S5 File CONSORT. (DOCX)

S6 File English version of the protocol for this trial. (PDF)

S7 File Protocol published in Medicine (Journal). (PDF)

S8 File Complete clinical protocol sent to the Ethics Committee in Research (translated to English). (PDF)

S9 File Complete clinical protocol sent to the Ethics Committee in Research (Original Portuguese). (PDF)

S10 File Protocols.io ELISA IL-10. (PDF)

S11 File Protocols.io GRIESS TEST. (PDF)
