# Supplementary material for: Effects of photobiomodulation on interleukin-10 and nitrites in individuals with relapsing-remitting multiple sclerosis – Randomized clinical trial
Source: PLoS One. 2020 Apr 7;15(4):e0230551. doi: 10.1371/journal.pone.0230551 (PMC7138327; doi:10.1371/journal.pone.0230551)
Supplement: S1 File — (PDF) [file pone.0230551.s001.pdf]

PARECER CONSUBSTANCIADO DO CEP

DADOS DA EMENDA

Título da Pesquisa: A efetividade da fotobiomodulação em indivíduos com Esclerose Múltipla - Ensaio clínico, controlado, aleatorizado

Pesquisador: TAMIRIS DA SILVA

Área Temática:

Versão: 4

CAAE: 72551717.0.0000.5511

Instituição Proponente: ASSOCIACAO EDUCACIONAL NOVE DE JULHO

Patrocinador Principal: ASSOCIACAO EDUCACIONAL NOVE DE JULHO

DADOS DO PARECER

Número do Parecer: 2.423.755

Apresentação do Projeto:

A esclerose múltipla (EM) é uma desordem inflamatória que se caracteriza por destruir de forma seletiva a bainha de mielina. Etiologia é multifatorial complexa e não é totalmente compreendida, acredita-se que formação de lesões desmielinizantes pode ser devido aos processos auto-imunes, mas também um resultado comum dos fatores ambientais e genéticos. Além disso, algumas evidências relatam um papel importante do óxido nítrico (ON) na patogênese da EM contribuindo com a inflamação, lesões de oligodendrócitos, alteração nas sinapses, degeneração axonal e morte neural.5 O ON é um radical livre bioativo que desempenha papel de neurotransmissor e neuromodulador do SNC.

Objetivo da Pesquisa:

Objetivo Primário: Avaliar a eficácia da fotobiomodulação nas regiões sublingual e medular em indivíduos com Esclerose Múltipla.

Objetivos secundários:

Avaliar a funcionalidade através o EDSS em indivíduos com Esclerose Múltipla;

Avaliar a expressão da IL-10, TNF- e ON

Comparar a fotobiomodulação na região sublingual e medular em pacientes com esclerose múltipla.

Avaliar se a fotobiomodulação possui efeito sistêmico (objetivo acrescentado nesta emenda)

Avaliação dos Riscos e Benefícios:

Os riscos esperados são mínimos, pois o participante será avaliado e receberá o tratamento em um local fechado, com a presença somente de um familiar (se necessário) e do pesquisador para evitar qualquer tipo de constrangimento. O pesquisador acompanhará o participante em todo trajeto a fim de evitar possíveis quedas. Explicará detalhadamente cada avaliação em que o indivíduo participará e o tratamento que receberá, posicionando-o com cuidado. O pesquisador permanecerá durante toda a avaliação e a aplicação do laser com luvas descartáveis. A pessoa que coletará o seu sangue é habilitada a utilizar os procedimentos adequados para não haver riscos para o(a) sr(a). Entretanto, observamos que há a possibilidade de ocorrer riscos e desconfortos relacionados à coleta venosa, ainda que raros e passageiros, como dor localizada. Raramente desmaio ou infecções no local de punção podem ocorrer. Cuidados devem ser tomados para minimizar esses riscos.

Os riscos e benefícios estão bem descritos e adequados.

Comentários e Considerações sobre a Pesquisa:

Pesquisa pertinente.

Considerações sobre os Termos de apresentação obrigatória:

Apresenta todos os termos adequados para a pesquisa

Recomendações:

Recomendação incluir no TCLE a faixa etária da população a ser estudada.

Conclusões ou Pendências e Lista de Inadequações:

Trata-se de um projeto aprovado pelo comitê (2.313.864), mas que solicita aprovação de emenda, neste caso acrescentando mais um objetivo: "Avaliar se a fotobiomodulação possui efeito sistêmico" . No projeto completo a metodologia está bem descrita e adequada.

O termo de consentimento foi modificado parcialmente com as informações referentes as avaliações, necessitando a inclusão da faixa etária da população a ser estudada.

Considerações Finais a critério do CEP:

Para início da coleta dos dados, o pesquisador deverá se apresentar na mesma instância que

autorizou a realização do estudo (Coordenadoria, Supervisão, SMS/Gab, etc). O sujeito de pesquisa (ou seu representante) e o pesquisador responsável deverão rubricar todas as folhas do Termo de Consentimento Livre e Esclarecido - TCLE apondo sua assinatura na última página do referido Termo, conforme Carta Circular no 003/2011 da CONEP/CNS. Salientamos que o pesquisador deve desenvolver a pesquisa conforme delineada no protocolo aprovado. Eventuais modificações ou emendas ao protocolo devem ser apresentadas ao CEP de forma clara e sucinta, identificando a parte do protocolo a ser modificada e suas justificativas. Lembramos que esta modificação necessitará de aprovação ética do CEP antes de ser implementada.

Ao pesquisador cabe manter em arquivo, sob sua guarda, por 5 anos, os dados da pesquisa,

contendo fichas individuais e todos os demais documentos recomendados pelo CEP (Res. CNS 466/2012).

De acordo com a Res. CNS 196, IX.2.c, o pesquisador deve apresentar a este CEP/SMS os relatórios semestrais. O relatório final deverá ser enviado através da Plataforma Brasil, ícone Notificação. Uma cópia digital (CD/DVD) do projeto finalizado deverá ser enviada à instância que autorizou a realização do estudo, via correio ou entregue pessoalmente, logo que o mesmo estiver concluído.

Este parecer foi elaborado baseado nos documentos abaixo relacionados:

Tipo Documento	Arquivo	Postagem	Autor	Situação
Informações Básicas do Projeto	PB_INFORMAÇÕES_BÁSICAS_1033277_E1.pdf	16/11/2017 14:51:20		Aceito
Projeto Detalhado / Brochura Investigador	Projeto.docx	16/11/2017 14:26:50	TAMIRIS DA SILVA	Aceito
Outros	emenda.docx	15/11/2017 09:24:10	TAMIRIS DA SILVA	Aceito
TCLE / Termos de Assentimento / Justificativa de Ausência	TCLE.docx	13/11/2017 20:02:39	TAMIRIS DA SILVA	Aceito
Folha de Rosto	Tamiris.pdf	20/06/2017 14:21:08	TAMIRIS DA SILVA	Aceito

Situação do Parecer:

Aprovado

Necessita Apreciação da CONEP:

Não

SAO PAULO, 07 de Dezembro de 2017

Assinado por:
Andrey Jorge Serra
(Coordenador)

Endereço: VERGUEIRO nº 235/249
Bairro: LIBERDADE **CEP:** 01.504-001
UF: SP **Município:** SAO PAULO
Telefone: (11)3385-9197 **E-mail:** comitedeetica@uninove.br
