# Supplementary material for: Effects of photobiomodulation on interleukin-10 and nitrites in individuals with relapsing-remitting multiple sclerosis – Randomized clinical trial
Source: PLoS One. 2020 Apr 7;15(4):e0230551. doi: 10.1371/journal.pone.0230551 (PMC7138327; doi:10.1371/journal.pone.0230551)
Supplement: S2 File — (PDF) [file pone.0230551.s002.pdf]

CONSUBSTANCED OPINION OF THE CEP

Researcher: TAMIRIS DA SILVA

Search title: The effectiveness of photobiomodulation in individuals with Multiple Sclerosis – Assay clinical, controlled, randomized

Institution: ASSOCIACAO EDUCACIONAL NOVE DE JULHO

Version: 4

CAAE: 72551717.0.0000.5511

DATA OF THE OPINION

Opinion Number: 2.423.755

Project presentation:

Multiple sclerosis (MS) is an inflammatory disorder characterized by selectively sheath of myelin. Etiology is multifactorial complex and not fully understood, it is believed that the formation of demyelinating lesions may be due to autoimmune processes, but also a result of environmental and genetic factors. In addition, some evidence important role of nitric oxide (ON) in the pathogenesis of MS contributing to inflammation, oligodendrocytes, synapse changes, axonal degeneration, and neural death.5 ON is a free radical bioactive agent that plays the role of neurotransmitter and neuromodulator of the CNS.

Search Objective:

Primary Objective: To evaluate the efficacy of photobiomodulation in sublingual and medullary regions in individuals with Multiple Sclerosis.

Secondary objectives:

Evaluate the functionality through the EDSS in individuals with Multiple Sclerosis;

To evaluate the expression of IL-10, TNF- and ON

To compare the photobiomodulation in the sublingual and medullar region in patients with multiple sclerosis.

To evaluate if the photobiomodulation has a systemic effect (objective added in this amendment).

Risk and Benefit Assessment:

The expected risks are minimal because the participant will be evaluated and receive the treatment in a place closed, with the presence of only one relative (if necessary) and the researcher to avoid any type of embarrassment. The researcher will accompany the participant in every way in order to avoid possible falls. It will explain in detail each evaluation in which the individual will participate and the treatment receive it, positioning it carefully. The researcher will remain throughout the evaluation and application with disposable gloves. The person who will collect your blood is entitled to use the procedures to avoid risk to you. However, we possibility of occurring risks and discomforts related to the venous collection, although rare and passengers, as localized pain. Rarely fainting or puncture site infections may occur. Care should be taken to minimize these risks. The risks and benefits are well described and adequate.

Comments and Research Considerations: Relevant research.

Considerations Regarding Mandatory Filing Terms: Displays all search terms.

Recommendations:

Recommendation to include in the TCLE the age range of the population to be studied.

Conclusions or Pending and List of Inadequacies: This is a project approved by the committee (2.313.864), but requests amendment approval in this if adding another objective: "Evaluate if the photobiomodulation has a systemic effect". In the project methodology is well described and adequate. The consent term was partially modified with the information regarding the evaluations, necessitating the inclusion of the age group of the population to be studied.

Final Considerations at CEP's discretion: To start collecting the data, the researcher must appear in the same instance that authorized the study (Coordination, Supervision, SMS / Gab, etc). The subject of research (or his representative) and the researcher in charge should initial all the leaves of the Term of Consent Free and Clarified - TCLE affixing its signature on the last page of said Term, according to Letter Circular no. 003/2011 of CONEP / CNS. We emphasize that the researcher must develop the research as outlined in the approved protocol. Any modifications or amendments to the protocol should be presented to the CEP in a clear and succinct way, identifying the part of the protocol to be modified and its justifications. We remind you that this modification will require the CEP's ethical approval before implemented. To the researcher it is necessary to keep in archives, under his guard, for 5 years, the data of the research, containing individual records and all other documents recommended by the CEP (Res. CNS 466/2012). According to Res. CNS 196, IX.2.c, the researcher must submit to this CEP / SMS the reports semesters. The final report should be sent through the Brazil Platform, Notification icon. A copy digital (CD / DVD) of the finished project should be sent to the instance that authorized the study, by mail or delivered personally, as soon as it is completed.

Status of Opinion: Approved

Needs Assessment of CONEP: Not

SAO PAULO, December 7, 2017.

Signed by: Andrey Jorge Serra (Coordinator)
