# Supplementary material for: Effects of photobiomodulation on interleukin-10 and nitrites in individuals with relapsing-remitting multiple sclerosis – Randomized clinical trial
Source: PLoS One. 2020 Apr 7;15(4):e0230551. doi: 10.1371/journal.pone.0230551 (PMC7138327; doi:10.1371/journal.pone.0230551)
Supplement: S3 File — (PDF) [file pone.0230551.s003.pdf]

TCLE - Termo de Consentimento para Participação em Pesquisa Clínica:

Nome do Voluntário:____
Endereço:____
Telefone para contato:__________________Cidade:________________CEP:________
E-mail: ____

1.Título do Trabalho Experimental: A efetividade da fotobiomodulação em indivíduos com Esclerose Múltipla após aplicação na medula espinal e sublingual – Ensaio clínico, controlado e aleatorizado.

2.Objetivo: Avaliar a eficácia da aplicação do laser em baixa intensidade em baixo da língua e na medula espinal em indivíduos com Esclerose Múltipla. Avaliar se a aplicação do laser poderá alterar os níveis óxido nítrico, TNF alfa (substancias inflamatórias) IL-10(Substância anti-inflamatória) e a escala EDSS.

3.Justificativa: Este estudo justifica-se uma vez que se verifica poucos estudos referente ao tratamento com o laser em baixa intensidade em indivíduos com esclerose múltipla.

4. Procedimentos da Fase Experimental: Todos os participantes passarão por consulta médica no ambulatório da UNINOVE, para confirmar o diagnóstico de esclerose múltipla. Através das amostras de 10 ml de sangue será realizado avaliação da IL-10 (anti-inflamatório), TNF alfa e o óxido nítrico (inflamatório), a coleta de sangue será um procedimento rápido de até 5 minutos, isso é, para avaliar função renal, hepática, e inflamação, a coleta de sangue será realizado pela equipe da enfermagem. Os participantes serão avaliados por meio da Escala de Status de Expansão Expandida (EDSS) que será aplicada em 15 minutos. Estas avaliações serão realizadas pré e após tratamento com o laser. Após as avaliações os participantes serão separados em 6 grupos sendo que o grupo 1 receberá um tratamento fictício com laser em baixo da língua, grupo 2 receberá tratamento real com laser em baixo da lingua, grupo 3 o participante receberá tratamento fictício do laser na região da medula, o grupo 4 receberá tratamento real com laser na região da medula, grupo 5 receberá o tratamento fictício na região da artéria radial (pulso), e grupo 6 receberá o tratamento real na região da artéria radial (pulso). É importante ressaltar que os participantes de todos os grupos devem continuar fazendo o tratamento medicamentoso. Para a aplicação do laser em baixo da língua, será utilizado um material plástico descartável cobrindo a caneta de aplicação, por questões de higiene Todos os participantes serão sorteados para fazer parte do grupo que receberá o tratamento real e o grupo que receberá o tratamento fictício. Se houver melhora nos grupos de tratamento com o laser, todos os participantes que forem sorteados para ficarem no grupo do tratamento fictício, receberão o tratamento real com a fotobiomodulação imediatamente após o término do estudo, para que não fique em desvantagem em relação ao outro grupo. Após o tratamento haverá acompanhado por 3 meses, e será necessário realizar novamente as avaliações.

5.**Desconforto ou Riscos Esperados:** Os riscos esperados são mínimos, pois o participante será avaliado e receberá o tratamento em um local fechado, com a presença somente de um familiar (se necessário) e do pesquisador para evitar qualquer tipo de constrangimento. O pesquisador acompanhará o participante em todo trajeto a fim de evitar possíveis quedas. Explicará detalhadamente cada avaliação em que o indivíduo participará e o tratamento que receberá, posicionando-o com cuidado. O pesquisador permanecerá durante toda a avaliação e a aplicação do laser com luvas descartáveis. A pessoa que coletará o seu sangue é habilitada a

utilizar os procedimentos adequados para não haver riscos para o(a) sr(a). Entretanto, observamos que há a possibilidade de ocorrer riscos e desconfortos relacionados à coleta venosa, ainda que raros e passageiros, como dor localizada. Raramente desmaio ou infecções no local de punção podem ocorrer. Cuidados devem ser tomados para minimizar esses riscos.

6. Retirada do Consentimento: Retirada do Consentimento: o voluntário tem a liberdade de retirar seu consentimento a qualquer momento e deixar de participar do estudo.

7. Informações: O participante tem a garantia que receberá respostas a qualquer pergunta ou esclarecimento de quaisquer dúvidas quanto aos procedimentos, riscos, benefícios e outros assuntos relacionados com a pesquisa. Também os pesquisadores citados assumem o compromisso de proporcionar informação atualizada obtida durante o estudo, ainda que esta possa afetar a vontade do indivíduo em continuar participando.

8. Garantia do Sigilo: Os pesquisadores asseguram a privacidade dos participantes quanto aos dados confidenciais envolvidos na pesquisa.

9. Formas de Ressarcimento das Despesas decorrentes da Participação na Pesquisa: Não serão ressarcidas despesas com eventuais deslocamentos.

10. Local da Pesquisa: A pesquisa será desenvolvida nas Clínicas de Fisioterapia da UNINOVE, localizadas nos Campus do Memorial (Rua Dr. Adolfo Pinto, 109 – Barra Funda), Campus da Vila Maria (Rua Profa. Maria José Barone Fernandes, 300 – Vila Maria) e no campus Vergueiro (Rua Vergueiro, 235/249 – Liberdade), São Paulo - SP, Brasil.

11. Comitê de Ética em Pesquisa (CEP) é um colegiado interdisciplinar e independente, que deve existir nas instituições que realizam pesquisas envolvendo seres humanos no Brasil, criado para defender os interesses dos participantes de pesquisas em sua integridade e dignidade e para contribuir no desenvolvimento das pesquisas dentro dos padrões éticos (Normas e Diretrizes Regulamentadoras da Pesquisa envolvendo Seres Humanos – Res. CNS nº 466/12). O Comitê de Ética é responsável pela avaliação e acompanhamento dos protocolos de pesquisa no que corresponde aos aspectos éticos.
Endereço do Comitê de Ética da Uninove: Rua. Vergueiro nº 235/249 – 12º andar - Liberdade – São Paulo – SP CEP. 01504-001 Fone: 3385-9197
comitedeetica@uninove.br

12. Nome Completo e telefones dos Pesquisadores (Orientador e Alunos) para Contato: : Profa. Dra. Sandra Kalil Bussadori (11) 98381-7453 ou Tamiris da Silva (11) 98737-6103.

13. Eventuais intercorrências que vierem a surgir no decorrer da pesquisa poderão ser discutidas pelos meios próprios.

São Paulo, de de .

15. Consentimento Pós-Informação:

Eu, ____, após leitura e compreensão deste termo de informação e consentimento, entendo que minha participação é voluntária, e que posso sair a qualquer momento do estudo, sem prejuízo algum. Confirmo que recebi uma via deste termo de consentimento, e autorizo a realização do trabalho de pesquisa e a divulgação dos dados obtidos somente neste estudo no meio científico.

Nome (por extenso):____

____
Assinatura do Participante / Responsável

16. Eu, ____ (Pesquisador do responsável desta pesquisa), certifico que:

a) Considerando que a ética em pesquisa implica o respeito pela dignidade humana e a proteção devida aos participantes das pesquisas científicas envolvendo seres humanos;
b) Este estudo tem mérito científico e a equipe de profissionais devidamente citados neste termo é treinada, capacitada e competente para executar os procedimentos descritos neste termo;
c) A resolução CNS nº 466/12 dispõe sobre as normas aplicáveis a pesquisas em Ciências Humanas e Sociais, cujo procedimentos metodológicos envolvam a utilização de dados diretamente obtidos com os participantes.

____
Tamiris da Silva
Assinatura do Pesquisador Responsável

1ª via: Instituição

2ª via: Voluntário
