# Supplementary material for: Effects of photobiomodulation on interleukin-10 and nitrites in individuals with relapsing-remitting multiple sclerosis – Randomized clinical trial
Source: PLoS One. 2020 Apr 7;15(4):e0230551. doi: 10.1371/journal.pone.0230551 (PMC7138327; doi:10.1371/journal.pone.0230551)
Supplement: S4 File — (PDF) [file pone.0230551.s004.pdf]

Declaration of consent for participation in clinical research:

Name of volunteer ____
Address:____
Telephone ________________ City:_______________ Postal code:___________
E-mail: ____

1. Title of experimental study: Effectiveness of photobiomodulation treatment in the sublingual region and along the spinal column in individuals with multiple sclerosis: Randomized, controlled, double-blind, clinical trial.

2. Objective: Evaluate the effectiveness of low-level laser administered under the tongue and along the spinal column in individuals with multiple sclerosis in terms of altering levels of nitric oxide, TNF alpha (proinflammatory substances), IL-10 (anti-inflammatory substance) and the results of the Expanded Disability Status Scale.

3. Justification: This study is justified by the fact that few studies have investigated the effectiveness of photobiomobulation in individuals with multiple sclerosis.

4. Procedures of experimental phase: All participants will go through a medical consultation at the UNINOVE clinic, to confirm the diagnosis of MS, and after which it will be collected by a nurse blood samples (10 ml), will also be taken for the determination of IL-10 (anti-inflammatory substance), TNF alpha and nitric oxide (proinflammatory substances). Blood collection is a fast process that takes less than five minutes. The evaluations will be performed before and after the treatment protocols (low-level laser administered along the spinal column and under the tongue). After the initial evaluation, the participants will be separated into 6 groups. Group 1 will receive fictitious laser treatment under the tongue. Group 2 will received active laser treatment under the tongue. Group 3 will receive fictitious laser treatment at points along the spinal cord and Group 4 will receive active treatment at points along the spinal cord. group 5 will receive the fictitious treatment in the region of the radial artery (pulse), and group 6 will receive the real treatment in the region of the radial artery (pulse). All participants in all groups should continue taking their regular medications. For laser administered under the tongue, the tip of the laser device will be covered with disposable plastic wrap for the purposes of hygiene. The participants will be selected using a random lottery process to determine to which group (real or fictitious, under the tongue or along the spinal cord) each participant will be allocated. If improvements are found in the groups submitted to active laser, all participants in the fictitious treatment groups will receive active photobiomodulation immediately after the end of the study so that no individuals are at a disadvantage in relation to the other group. After the treatment, it will be followed for 3 months, and it will be necessary to carry out the evaluations again.

5. **Discomfort and expected risks:** The expected risks are minimal. The participant will be evaluated and submitted to treatment in a closed environment with the presence of a family member (if necessary) and the researcher will avoid any type of embarrassment for the volunteer. The researcher will accompany the participant throughout the entire visit to the clinic to avoid possible falls and will offer detailed explanations of the evaluation process and treatment to which the individual will be submitted. The volunteer will be positioned for treatment with the utmost care and the researcher will remain throughout the entire evaluation and low-level laser administration process, wearing disposable gloves. The nurse who will collect blood is skilled at using adequate procedures to minimize any risk. However, there is the possibility of risk and discomfort

related to collecting blood from the vein, although rare and fleeting, such as pain at the collection site. In rare instances, fainting or infection of the collection site may occur. Utmost care will be taken to minimize such risks.

6. Withdrawal of consent: The volunteer is free to withdraw consent at any time and decline to participate in the study.

7. Information: The participant will receive clarifications for any questions regarding the procedures, risks, benefits and other issues related to the study. The researchers also assume the commitment of providing updated information throughout the study, even if this may affect the participant’s willingness to continue participating.

8. Confidentiality: The researchers will ensure the participant’s privacy regarding the confidential data involved in the study.

9. Forms of reimbursement of expenses related to participation in study: No expenses on transportation costs related to the treatment appointments will be reimbursed.

10. Location of study: The study will be developed at the Physiotherapy Clinics of UNINOVE located on the Memorial Campus (109 Dr. Adolfo Pinto Street in the neighborhood of Barra Funda), the Vila Maria Campus (300 Profa. Maria José Barone Fernandes Street in the neighborhood of Vila Maria) and Vergueiro Campus (235/249 Vergueiro Street in the neighborhood of Liberdade) in the city of São Paulo, SP, Brazil.

11. The Human Research Ethics Committee is an independent, interdisciplinary review board that must exist in institutions that perform research involving human subjects in Brazil. It was created to defend the interests, integrity and dignity of participants in studies and contribute to the development of studies conducted in compliance with ethical standards (Regulating Norms and Guidelines for Research Involving Human Subjects – Resolution nº 466/12 of the Brazilian National Board of Health). The ethics committee is responsible for the evaluation and accompaniment of study protocols with regard to ethical aspects.
Address of UNINOVE Ethics Committee: Vergueiro Street nº 235/249, 12th floor – neighborhood of Liberdade, São Paulo, SP, Postal code: 01504-001 Telephone: 3385-9197 email: comitedeetica@uninove.br

12. Name and telephone of researchers (adviser and student): Prof. Dr. Sandra Kalil Bussadori (11) 98381-7453 and Tamiris da Silva (11) 98737-6103.

13. Any complications that arise during the study will be resolved through the proper channels.

São Paulo, (date) ______________________ .

15. Post-information consent:

I, ____, after reading and understanding this statement of information and consent, understand that my participation is voluntary and I can leave the study at any time with no negative consequences. I declare that I have received a copy of this statement of informed consent. I authorize the execution of the study and divulgation of the data obtained only in this study to the scientific community.

Name (printed):____

____
Signature of participant or guardian

16. I, ____ (researcher in charge of study), certify that:

a) Considering that ethics in research implies respect for human dignity and protection to the participants of scientific research involving human subjects;
b) This study has scientific merit and the researchers cited in this statement are trained and competent for the execution of the procedures described herein;
c) Resolution nº 466/12 of the National Board of Health stipulates the norms applicable to studies in the human and social sciences the procedures of which involve the use of data obtained directly from the participants.

____
Tamiris da Silva
Signature of chief researcher

1st copy: Institution

2nd copy: Volunteer
