# Supplementary material for: Effects of photobiomodulation on interleukin-10 and nitrites in individuals with relapsing-remitting multiple sclerosis – Randomized clinical trial
Source: PLoS One. 2020 Apr 7;15(4):e0230551. doi: 10.1371/journal.pone.0230551 (PMC7138327; doi:10.1371/journal.pone.0230551)
Supplement: S6 File — (PDF) [file pone.0230551.s006.pdf]

Effect of photobiomodulation treatment in the sublingual, radial artery region, and along the spinal column in individuals with multiple sclerosis: Protocol for a randomized, controlled, double-blind, clinical trial

Abstract

Background: Multiple sclerosis (MS) is an autoimmune disease, for which the forms of treatment are medication and rehabilitation. However, *in vitro* and *in vivo* studies have demonstrated that photobiomodulation can be an effective treatment modality for inflammatory diseases, including MS. Photobiomodulation has a broad range of benefits, such as the avoidance of cell and tissue death, the stimulation of healing and injury repair, reductions in pain, edema and inflammation, cell proliferation and even apoptosis. The outcomes of photobiomodulation include the regeneration of cells, the stimulation of the growth of Schwann cells, a reduction in spasticity, functional improvements, a reduction in nitric oxide levels and the upregulation of the cytokine IL10, demonstrating that this therapeutic modality can offer neuro-protection.

Methods: A randomized, controlled, double-blind, clinical trial is proposed. The patients will be divided into 6 groups. Groups 1 and 2 will receive sham and active photobiomodulation in the sublingual region, respectively. Groups 3 and 4 will receive sham and active photobiomodulation along the spinal cord, respectively. Group 5 will receive placebo treatment with photobiomodulation on the skin in the region of the radial artery with a specific bracelet. Group 6 will be treated with photobiomodulation on the skin in the region of the radial artery.

Discussion: Treatment for MS is directed at the immune response and slowing the progression of the disease. This is one of the first clinical trials involving photobiomodulation in the sublingual region and along the spinal cord, which could help establish a promising new form of non-pharmacological treatment for autoimmune diseases. This is one of the first clinical trials with sublingual photobiomodulation and along

the spinal cord that could help establish a new form of promising treatment of the disease associated with pharmacological treatment.

Key words: Low-level laser therapy, photobiomodulation, multiple sclerosis, oxidative stress, physical therapy.

1. Introduction

Multiple sclerosis (MS) is an demyelinating, neurodegenerative, inflammatory disorder of the central nervous system (CNS) characterized by the selective destruction of the myelin sheath.[1,2] MS has a complex, multifactor etiology that is not fully understood, but it is believed that the formation of demyelinating lesions may be due to autoimmune processes as well as environmental and genetic factors.[3,4]

Inflammation of the CNS is an important mechanism that contributes to demyelination and neurodegeneration. Th1, Th17, and B cells are activated in peripheral regions, pass through the blood–brain barrier, and interact with antigen-presenting cells (astrocytes, microglia, macrophages, and dendritic cells), inducing the production of proinflammatory cytokines and oxidative stress.[5,6] B lymphocytes serve as specific antigen-presenting cells for T cells and produce specific antibodies for myelin antigens, making myelin the target of immune cells that mistake it for a foreign antigen.[6,7]

Oxidative stress occurs due to the accumulation of free radicals (reactive oxygen and nitrogen species),[8] which leads to inflammation, oligodendrocyte damage, abnormalities in synaptic transmissions, axonal degeneration, and neuronal death, suggesting that oxidative stress is an important factor in neurodegeneration.[9,10] Myelin and oligodendrocyte damage caused by inflammation gives rise to a multitude of symptoms, such as sensory alterations, fatigue, physical and/or mental disability, balance disorders, spasticity, muscle weakness, urinary incontinence, cognitive impairment, neuropathic pain, and visual deficiency.[1,3]

This disease occurs in different forms, such as relapsing–remitting MS, which is characterized by sudden-onset short-term or long-term relapses, secondary progressive MS, which has a progressive course that results in severe, irreversible disability, and primary progressive MS, which is a progressive type with no relapses or periods of remission.[1,11,12]

Prognosis is unpredictable with regard to the disability that occurs due to the manifestations of the disease, which are normally associated with progressive locomotion impairment.[12] Treatment is directed at the immune response and slowing the progression of the disease, which can be achieved with the use of drugs.[12,13] Moreover, rehabilitation can lead to improvements in walking capacity, cognition, fatigue, depression, quality of life, participation in activities, muscle strength, cardiovascular

performance, and balance.[13,14]

In vitro and in vivo studies have demonstrated that photobiomodulation is effective for inflammatory diseases, including MS.[15] This therapeutic modality has a broad range of benefits, such as the avoidance of cell and tissue death, the stimulation of healing and injury repair, reductions in pain, edema and inflammation, cell proliferation, and even apoptosis.[15,16] The outcomes of photobiomodulation include the regeneration of

cells, the stimulation of the growth of Schwann cells, a reduction in spasticity, functional improvements, a reduction in nitric oxide levels, and the upregulation of the cytokine IL10, demonstrating that this therapeutic modality can offer neuroprotection.[15,17]

The aim of the proposed study is to evaluate whether photobiomodulation in the sublingual region and along the spinal cord modulates the expression of IL-10, TNF-a,

and nitric oxide in individuals with MS. The main objective of this study is to evaluate if the photobiomodulation along the spinal cord in the sublingual region and irradiation in the radial artery can modulate the expression of IL-10, TNF-a, and nitric oxide.

Methods/Design

Trial design

A controlled, clinical trial is proposed, which will follow the guidelines for research involving human subjects stipulated in Resolution 466/2012 of the Brazilian National Board of Health and will be submitted for approval from the Human Research Ethics Committee of University Nove de Julho. The participants or their legal guardians will sign statements of informed consent authorizing participation in the study (APPENDIX 1).

Inclusion criteria: Diagnosis of MS, age between 18 and 60 years, currently undergoing pharmacological treatment, capable of understanding and following verbal instructions and score of < 7 on the Expanded Disability Status Scale. No restriction will be imposed regarding gender.

Exclusion criteria: other autoimmune disease and/or tumor, relapse of disease activity during treatment, and not undergoing pharmacological treatment. Other autoimmune diseases; Neoplasias, Heart failure, Respiratory failure, Renal insufficiency, Hepatic insufficiency, Acquired immunodeficiency syndrome, Patients with relapses of the disease.

Recruitment and randomization

Patients will be recruited from the Integrated Health Clinic of University (Vergueiro, Memorial and Vila Maria campuses). Patients with a diagnosis of MS will be screened for the eligibility criteria through telephone interviews. Selected individuals with a signed statement of informed consent will be randomized. Randomization will be performed in blocks. Groups 1 and 2 will receive sham and active photobiomodulation in the sublingual region, respectively. Groups 3 and 4 will receive sham and active photobiomodulation along the spinal cord, respectively. Group 5 will receive placebo treatment with photobiomodulation on the skin in the region of the radial artery with a specific bracelet. Group 6 will be treated with photobiomodulation on the skin in the region of the radial artery. The participants will not be deprived of any medication for the treatment of the

base condition. Randomization will be stratified by clinic using block allocation tables to ensure equal proportions in the distribution of treatments. The participants and evaluator will be blinded to the allocation.

Evaluations

EDSS

The participants will be evaluated before and after treatment using the Expanded Disability Status Scale administered by a physiotherapist (approximate application time: 15 minutes).

Blood collection for analyzes of inflammation, oxidative stress

All participants will go through a medical consultation at the UNINOVE clinic, to confirm the diagnosis of MS, and after which it will be collected by a nurse blood samples (10 ml) will also be taken for the determination of IL-10 (anti-inflammatory), TNF alpha and nitric oxide (proinflammatory). The evaluation of these cytokines will be through ELISA and griess reaction.

Treatment

The participant will be placed on an examining table in a comfortable position. Both the operator and participant will use eye protection. Transcutaneous irradiation of the spinal cord will be performed on segments corresponding to the nerve roots of the lumbosacral plexus (T12-S5) and cervicothoracic plexus (C5-T1-2). Twenty points will be irradiated for 90seconds (total treatment time: 1800seconds). In the group submitted to sublingual irradiation, disposable plastic wrap will cover the application pen for the purposes of hygiene, (total treatment time: 360seconds). In the group submitted photobiomodulation on the skin in the region of the radial (total treatment time: 360seconds). The treatment will be performed twice a week totaling 24 consecutive weeks and after 3 months of treatment the patients will be submitted to reassessment of all complementary tests that have been requested, cytokines, nitric oxide, and EDSS.

Photobiomodulation protocol

With regard to the photobiomodulation protocols, the articles of interest were identified through a bibliographic survey of titles and abstracts. The selected articles were analyzed and used for the establishment of the treatment protocol (TABLE 1).

Table1

PARAMETER	UNIDADE	SPINAL CORD	SUBLINGUAL	ARTÉRIA RADIAL
CENTER WAVELENGTH	nm	808	808	808
DIAMETRO	cm	0,4	0,4	0,4
AREA	Cm^2	0,1256	0,1256	0,1256
IRRADIANCIA	$(W/cm^2$	1433	287	287
TIME	s	1800	360	360
ENERG	J	179,9848	36,0472	36,0472

Statistical analysis

The data will be tabulated and treated using the SPSS 20.0 for Windows. Descriptive statistics will be performed. The chi-square test and Fisher's exact test will be used to test associations with the categorical variables. The Student's t-test will be used and Pearson's correlation coefficients will be calculated for the analysis of correlations among the continuous variables. The level of significance will be set at 95% ($p < 0.05$).

Sample calculation

The sample size was calculated to ensure a 95% test power. It was determined that with 32 individuals and an effect size of 0.8, the test power would be 0.9566, thereby maintaining the significance level at $\alpha = 0.05$ (Figure 1).

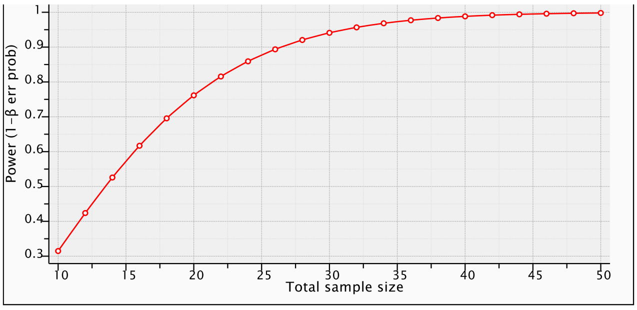

Figure 1

Discussion

Treatment for MS is directed at the immune response and slowing the progression of the disease. This is one of the first clinical trials involving photobiomodulation in the sublingual region and along the spinal cord, which could help establish a promising new form of non-pharmacological treatment for autoimmune diseases.

References

[1] Ransohoff RM, et al. Multiple sclerosis—a quiet revolution. Rev Neurol. 2015;11:134–42.

[2] Albarracín JDF, Segura OME. Esclerosis multiple en pacientes pediátricos: fisiopatología, diagnóstico y manejo. Rev Med UNABI Vol 2012;14:167–79.

[3] Kubsik A, et al. Application of laser radiation and magnetostimulation in therapy of patients with multiple sclerosis. NeuroRehabilitation 2016;183–90.

[4] Deckx N, et al. 2 Weeks of Combined Endurance and Resistance Training Reduces Innate Markers of Inflammation in a Randomized Controlled Clinical Trial in Patients with

Multiple Sclerosis. Mediators of InflammationVolume 2016, Article ID 6789276, 13 pages. http://dx. doi.org/10.1155/2016/6789276.

[5] Murphy AC, Lalor SJ, Lync , et al. Infiltration of Th1 and Th17 cells and activation of microglia in the CNS during the course of experimental autoimmune encephalomyelitis. Brain Behav Immun. 2010;24:641–51.

[6] Pilli D, Zou A, Tea F, et al. Expanding role of t cells in human autoimmune diseases of the central nervous system. Front Immunol. 2017;8:1–6.

[7] Lisak RP, Nedelkoska L, Benjamins JA, et al. B cells from patients with multiple sclerosis induce cell death via apoptosis in neurons in vitro. J Neuroimmunol 2017;309:88–99.

[8] Voigt D, Scheidt U, Derfuss T, et al. Expression of the antioxidative enzyme peroxiredoxin 2 in multiple sclerosis lesions in relation to inflammation. Int J Mol Sci 2017;18:760.

[9] Ghasemi M, Fatemi A. Pathologic role of glial nitric oxide in adult and pediatric neuroinflammatory diseases. Neurosci Biobehav Rev 2014;5:168–82.

[10] Calabrese V, Calabrese C, Rizzarelli E, et al. Nitric oxide in the central nervous system: neuroprotection versus neurotoxicity.Nat RevNeurosci 2007;8:766–75.

[11] Thompson AJ, Baranzini SE, Geurts J, et al.Multiple sclerosis. Published online March 22, 2018. http://dx.doi.org/10.1016/S0140-6736(18) 30481-1.

[12] Hempel S, et al. A systematic review of modifiable risk factors in the progression of multiple sclerosis. Mult Scler 2017;23:525–33.

[13] Alvarenga FH, Sacramento PM, Ferreira TB, et al. Combined exercise training reduces fatigue andmodulates the cytokine profile of Tcells from multiple sclerosis patients in response to neuromediators. J Neuroimmunol 2016;293:91–9.

[14] Learmonth YC, Adamson BC, Balto JM, et al. Investigating the needs and wants of healthcare providers for promoting exercise in persons with multiple sclerosis: a qualitative study. Disabil Rehabil 2017;19:1–9.

[15] Gonçalves DG, et al. Low-level laser therapy ameliorates disease progression in a mouse model of multiple sclerosis. Autoimmunity 2016;49:132–42.

[16] Muili KA, Gopalakrishnan S, Meyer SL, et al. Amelioration of experimental autoimmune encephalomyelitis in C57BL/6 mice by photobiomodulation induced by 670nm light. PLoS One 2012; 7:1–9.

[17] Muili KA, Gopalakrishnan S, Meyer SL, et al. Photobiomodulation induced by 670nm light ameliorates MOG35 induce EAE in female C57BL/6 mice: a role for remediation of nitrosative stress. PLoS One 2013;8:e67358.

APPENDIX I – Statement of informed consent

Declaration of consent for participation in clinical research:

Name of volunteer ____
Address:____
Telephone _________________ City:________________ Postal code:___________
E-mail: ____

1. Title of experimental study: Effectiveness of photobiomodulation treatment in the sublingual region and along the spinal column in individuals with multiple sclerosis: Randomized, controlled, double-blind, clinical trial.

2. Objective: Evaluate the effectiveness of low-level laser administered under the tongue and along the spinal column in individuals with multiple sclerosis in terms of altering levels of nitric oxide, TNF alpha (proinflammatory substances), IL-10 (anti-inflammatory substance) and the results of the Expanded Disability Status Scale.

3. Justification: This study is justified by the fact that few studies have investigated the effectiveness of photobiomobulation in individuals with multiple sclerosis.

4. Procedures of experimental phase: All participants will go through a medical consultation at the UNINOVE clinic, to confirm the diagnosis of MS, and after which it will be collected by a nurse blood samples (10 ml), will also be taken for the determination of IL-10 (anti-inflammatory substance), TNF alpha and nitric oxide (proinflammatory substances). Blood collection is a fast process that takes less than five minutes. The evaluations will be performed before and after the treatment protocols (low-level laser administered along the spinal column and under the tongue). After the initial evaluation, the participants will be separated into 6 groups. Group 1 will receive fictitious laser treatment under the tongue. Group 2 will received active laser treatment under the tongue. Group 3 will receive fictitious laser treatment at points along the spinal cord and Group 4 will receive active treatment at points along the spinal cord. group 5 will receive the fictitious treatment in the region of the radial artery (pulse), and group 6 will receive the real treatment in the region of the radial artery (pulse). All participants in all groups should continue taking their regular medications. For laser administered under the tongue, the tip of the laser device will be covered with disposable plastic wrap for the purposes of hygiene. Treatment will be performed in three weekly sessions over five consecutive weeks. The participants will be selected using a random lottery process to determine to which group (real or fictitious, under the tongue or along the spinal cord) each participant will be allocated. If improvements are found in the groups submitted to active laser, all participants in the fictitious treatment groups will receive active photobiomodulation immediately after the end of the study so that no individuals are at a disadvantage in relation to the other group. After the treatment, it will be followed for 3 months, and it will be necessary to carry out the evaluations again.

5. **Discomfort and expected risks:** The expected risks are minimal. The participant will be evaluated and submitted to treatment in a closed environment with the presence of a family member (if necessary) and the researcher will avoid any type of embarrassment for the volunteer. The researcher will accompany the participant throughout the entire visit to the clinic to avoid possible falls and will offer detailed explanations of the evaluation process and treatment to which the individual will be submitted. The volunteer will be positioned for treatment with the utmost care and the researcher will remain

throughout the entire evaluation and low-level laser administration process, wearing disposable gloves. The nurse who will collect blood is skilled at using adequate procedures to minimize any risk. However, there is the possibility of risk and discomfort related to collecting blood from the vein, although rare and fleeting, such as pain at the collection site. In rare instances, fainting or infection of the collection site may occur. Utmost care will be taken to minimize such risks.

6. Withdrawal of consent: The volunteer is free to withdraw consent at any time and decline to participate in the study.

7. Information: The participant will receive clarifications for any questions regarding the procedures, risks, benefits and other issues related to the study. The researchers also assume the commitment of providing updated information throughout the study, even if this may affect the participant's willingness to continue participating.

8. Confidentiality: The researchers will ensure the participant's privacy regarding the confidential data involved in the study.

9. Forms of reimbursement of expenses related to participation in study: No expenses on transportation costs related to the treatment appointments will be reimbursed.

10. Location of study: The study will be developed at the Physiotherapy Clinics of UNINOVE located on the Memorial Campus (109 Dr. Adolfo Pinto Street in the neighborhood of Barra Funda), the Vila Maria Campus (300 Profa. Maria José Barone Fernandes Street in the neighborhood of Vila Maria) and Vergueiro Campus (235/249 Vergueiro Street in the neighborhood of Liberdade) in the city of São Paulo, SP, Brazil.

11. The Human Research Ethics Committee is an independent, interdisciplinary review board that must exist in institutions that perform research involving human subjects in Brazil. It was created to defend the interests, integrity and dignity of participants in studies and contribute to the development of studies conducted in compliance with ethical standards (Regulating Norms and Guidelines for Research Involving Human Subjects – Resolution nº 466/12 of the Brazilian National Board of Health). The ethics committee is responsible for the evaluation and accompaniment of study protocols with regard to ethical aspects.
Address of UNINOVE Ethics Committee: Vergueiro Street nº 235/249, 12th floor – neighborhood of Liberdade, São Paulo, SP, Postal code: 01504-001 Telephone: 3385-9197 email: comitedeetica@uninove.br

12. Name and telephone of researchers (adviser and student): Prof. Dr. Sandra Kalil Bussadori (11) 98381-7453 and Tamiris da Silva (11) 98737-6103.

13. Any complications that arise during the study will be resolved through the proper channels.

São Paulo, (date) ______________________ .

15. Post-information consent:

I, ____, after reading and understanding this statement of information and consent, understand that my participation is voluntary and I can leave the study at any time with no negative consequences. I declare that I have received a copy of this statement of informed consent. I authorize the execution of the study and divulgation of the data obtained only in this study to the scientific community.

Name (printed):____

____
Signature of participant or guardian

16. I, ____ (researcher in charge of study), certify that:

a) Considering that ethics in research implies respect for human dignity and protection to the participants of scientific research involving human subjects;
b) This study has scientific merit and the researchers cited in this statement are trained and competent for the execution of the procedures described herein;
c) Resolution nº 466/12 of the National Board of Health stipulates the norms applicable to studies in the human and social sciences the procedures of which involve the use of data obtained directly from the participants.

____
Tamiris da Silva
Signature of chief researcher

1st copy: Institution

2nd copy: Volunteer
