# Supplementary material for: Effects of photobiomodulation on interleukin-10 and nitrites in individuals with relapsing-remitting multiple sclerosis – Randomized clinical trial
Source: PLoS One. 2020 Apr 7;15(4):e0230551. doi: 10.1371/journal.pone.0230551 (PMC7138327; doi:10.1371/journal.pone.0230551)
Supplement: S8 File — (PDF) [file pone.0230551.s008.pdf]

PHOTOBIOMODULATION EFFECTIVENESS IN INDIVIDUALS WITH MULTIPLE SCLEROSIS AFTER APPLICATION IN THE SPINAL AND SUBLINGUAL MARROW - CLINICAL, CONTROLLED, RANDOMIZED TEST.

São Paulo

2017

1. Problem Statement

1.1. Multiple sclerosis

Multiple sclerosis (MS) is a demyelinating and neurodegenerative inflammatory disorder of the central nervous system (CNS) that is characterized by selectively destroying the myelin sheath.[1,2] Its etiology is multifactorial, complex and not fully understood, however, it is believed that the formation of demyelinating lesions is due to autoimmune processes, and also a result of environmental and genetic factors.[3,4]

Although the precise mechanisms that trigger the disease have not been fully elucidated, it is known that Th1, Th17 and CD4 cells are dysregulated, they are activated in the periphery and migrate mainly through the blood-brain barrier (BBB) invading the CNS and interact with antigen presenting cells (CAA), namely astrocytes, microglia, macrophages and dendritic cells (DC), inducing the production of proinflammatory cytokines such as interleukin (IL) 17 and tumor necrosis factor alpha (TNF) - α). [5,6,7,8,9]

B lymphocytes act as T-cell-specific CAA cells, and when activated they also penetrate through BBB, and produce antibodies specific for myelin antigens, thus myelin becomes a target for immune cells, which recognizes it as a foreign antigen. and destroys it. [6,7,10]

In addition, some evidence reports an important role of oxidative stress in the pathogenesis of MS and its contribution to inflammation, oligodendrocyte injury, changes in synaptic transmission, axonal

degeneration, and neuronal death, suggesting that oxidative stress contributes to neurodegeneration.[5,11]

Oxidative stress occurs due to the accumulation of free radicals, ie reactive oxygen species (ROS) and nitrogen reactive species (RNS).[5,12] Recent studies report that there is a relationship between neurons and immune cells, which maintains and guarantees CNS homeostasis and the imbalance between free radical production and the antioxidant capacity of cells is responsible for neuronal damage.[5,6]

Under normal conditions, ROS and RNS alter proteins and damage DNA / RNA, however, CNS cells are able to protect against ROS and RNS by inactivating these highly reactive molecules and repairing emerging damage. This ability critically depends on the expression of antioxidant molecules. The CNS is particularly sensitive to oxidation due to its high oxygen consumption and the relatively low level of endogenous antioxidants.[5,6]

Nitric oxide (ON) is an NRA, and plays an important role in the pathogenesis of MS contributing to inflammation, oligodendrocyte damage, synapse alteration, axonal degeneration and neural death5. At low concentrations, it plays a physiological role in cells, including neurotransmission, regulation of blood vessel tone and immune response, and at higher concentrations, it is implicated in the pathogenesis of various neurological diseases such as neurodegenerative, demyelinating diseases and neuroinflammatory diseases.[13,14]

ON is enzymatically produced from the amino acid l-arginine through the synthesis activities of ON (NOS) enzymes. After degradation of these enzymes, three subtypes of NOS are identified, namely Neuronal NOS (nNOS or NOS1), Endothelial NOS (eNOS or NOS3) and Induced NOS (iNOS or NOS2. This latter type indicates that enzyme expression is induced iNOS is expressed in various cell types, especially glial cells, mainly astrocyte and microglia. The microglia expresses only iNOS under pathological conditions such as ischemia, trauma, neurotoxic and inflammatory damage including MS.[15,13, 16]

The microglia are resident in the CNS and physiologically perform a variety of tasks, including cell maintenance, release of trophic and anti-inflammatory factors that facilitate stem cell migration to the site of inflammation, and also have macrophage-like capabilities, including phagocytosis and inflammatory cytokine production.[15] However, when activated and triggers proinflammatory responses and alters the oxidant / antioxidant balance, leading to neuronal loss.[5,15,13]

Microglia and astrocytes represent an important source of neurotoxic molecules, which are the inflammatory medicaments, reactive oxygen species (ROS) and nitrogen (RNS), which are responsible for the progressive loss of neuronal cell structure and function.[5] The result of inflammation and oxidative stress is damage to myelin and oligodendrocytes, causing a multiplicity of symptoms.[7,18]

The initial symptoms are heterogeneous with variable progression over time, including sensory changes, fatigue, physical and / or mental

disability, balance disorders, spasticity, muscle weakness, urinary incontinence, cognitive impairment, neuropathic pain, visual disorders.[3,4,17, 19,20,21]

The disease manifests itself in a number of ways, such as: Recurrent Remitting (RR), which is characterized by sudden relapses punctuated by short or long term remissions; Secondarily Progressive (SP), which has a progressive course resulting in severe and irreversible debilitation and Primarily Progressive (PP), which is a progressive type of MS without initial recurrence and remission period.[17,19,22] In general, relapses are due to acute focal inflammation and disease progression is caused by chronic neurodegeneration.[7]

Diagnosis of MS in adults and children requires recurrent episodes of CNS demyelination, imaging findings and cerebrospinal fluid (CSF), elevated blood plasma levels of oxidative / nitrative damage proteins, which is important to provide information on inflammatory process, besides the clinical presentation.[6,23]

MS typically present between the ages of 20-40 years, but may manifest during childhood or adolescence. The overall incidence of childhood MS is unknown and few epidemiological studies show results, it is estimated that 3% to 10% of all MS patients will begin before 18 years of age.[1,24,25]

The prognosis is unpredictable in relation to the disability that occurs due to sequelae, usually associated with progressive impairment of locomotion21. Regarding children, despite an earlier

inflammatory course, initial recovery is better and disease progression is slower in children than in adults.[26]

Expanded Expansion Status Scale (EDSS) is a method for quantifying and monitoring the disability of individuals with MS. Higher EDSS scores correspond to higher disease severity clinically.[3,6,27]

Treatment can be performed with drugs, and rehabilitation that has shown improvements in walking, cognition, fatigue, depression, QoL, task participation, improves muscle strength, cardiovascular performance, mental functioning, balance, fatigue, and quality of life.[3, 4.28.29]

In vitro and in vivo studies have shown the efficacy of photobiomodulation (FBM) in inflammatory diseases, including deminielizing diseases such as MS.[6,16] FBM can modulate a broad spectrum of cellular processes, including: Prevent cell and tissue death, stimulation of wound healing and repair, pain reduction, edema and inflammation, proliferation to apoptosis.[15,17]

Studies have shown that FBM causes a sequence of changes at the cellular level, and the result is, among others, cell regeneration, including stimulation of Schwann cell growth, decreased spasticity, and functional improvement, as well as efficiently reducing blood cell levels. ON.[3,15,17] Photobiomodulation in the experimental model of autoimmune encephalopathy (EAE) resulted in a positive regulation of IL10, which is an anti-inflammatory cytokine showing that photobiomodulation can offer neuro protection in the EAE model.[11,30]

Recently, FBM was used to control inflammation, prevent joint damage or loss of function, and decrease pain and systemic injury in Juvenile Rheumatoid Arthritis (JRA). This study aimed to introduce a new technique and investigate the effects of FBM. sublingual in patients with JRA with different wavelengths.

Blood circulation is very abundant in the dorsal tongue of children, so laser irradiation of this area can bring great benefits, being noninvasive and painless. Results showed reduced pain, increased quality of life.[31]

1.2. Justification

Treatments for multiple sclerosis (MS) are for immune response and slow disease progression. In MS, inflammatory cytokines of Th1 are known to be present and there is a high concentration of Nitric Oxide, and in vivo and in vitro studies have shown that photobiomodulation can modulate nitric oxide levels. However, the literature has been scarce of studies on the subject, mainly controlled clinical trials and there are no studies showing the effect of sublingual photobiomodulation, and only 1 study was performed in humans where the laser was applied in the spinal cord region.[3]

Thus sublingual and spinal photobiomodulation may be a promising non-pharmacological disease therapy for the treatment of autoimmune conditions such as MS.

2. Objective

To evaluate the effectiveness of the spinal cord regions photobiomodulation, sublingual, and radial artery in subjects with Multiple Sclerosis.

2.1 Objetivos secundários

- Avaliar a funcionalidade através o EDSS em indivíduos com Esclerose Múltipla;
- Avaliar a expressão da IL-10, TNF- α e ON
- Comparar a fotobiomodulação na região sublingual e medular em pacientes com esclerose múltipla
- Avaliar se a fotobiomodulação possui efeito sistêmico

2.1 Secondary Objectives

- Evaluate functionality through EDSS in individuals with Multiple Sclerosis;
- Evaluate IL-10, TNF-α and ON expression
- Compare the photobiomodulation in sublingual and spinal region in patients with multiple sclerosis
- Evaluate if photobiomodulation has a systemic effect

3. Materials and methods

3.1. Study design

This study will be a randomized controlled clinical trial that will be developed according to the flowchart presented (FIGURE 1). It will follow the regulatory norms (resolution number 466/2012) in human research with submission and approval of the Research Ethics Committee of the Nove de Julho University. Participants or guardians will sign the Informed Consent (IC) and Consent (TA) to authorize participation in the research (ANNEX1).

The recruitment process will be at the Nove de Julho University (UNINOVE) Integrated Health Outpatient Clinic, Memorial Campus and Vila Maria. Patients diagnosed with MS will be screened by telephone and if they meet the inclusion criteria and give written informed consent should be randomized. Randomization will be stratified by center with tables using block allocation to provide equal treatment distribution. Participants and the evaluator will not know which groups they will be in.

10 ml blood samples will be collected by a nurse where oxidative stress (nitrite / nitrate), inflammatory mediators (TNF-α) will be evaluated and the anti-inflammatory effect of photobiomodulation (IL-10) will be investigated. The evaluation of these cytokines will be through the ELISA test and griess reaction. Participants' physical disabilities will also be assessed using the Expanded Expansion Status Scale (EDSS), which will be applied within 20 minutes.

After evaluation the participants will be divided into 6 groups: Group 1 will receive placebo treatment with sublingual photobiomodulation, group 2 will receive treatment with photobiomodulation in the sublingual region, Group 3 will receive placebo treatment with photobiomodulation in the spinal region, group 5 will receive treatment with photobiomodulation in the radial artery region, group 6 will receive placebo with photobiomodulation in the radial artery region.

Importantly, participants in all groups will not be deprived of any medication to treat their underlying condition. If there is improvement in the photobiomodulation treatment groups, all participants who are drawn to the dummy treatment group will receive the actual photobiomodulation treatment immediately after the end of the study so that they are not disadvantaged by the other. group.

Will be included in the research, individuals diagnosed with Multiple Sclerosis, EDSS up to 6, aged 18 to 60 years, and should be undergoing pharmacological treatment. Individuals with other autoimmune diseases, neoplasms, heart failure, respiratory failure, renal failure, liver failure, acquired immunodeficiency syndrome, and outbreaks of the disease will be excluded from the study.

3.3. Avaliações

3.3.1. Avaliação funcional - EDSS (Expanded Disability Status Scale)

A EM é considerada uma das doenças mais incapacitantes dentre as relacionadas ao SNC e a mensuração desta incapacidade funcional torna-se indispensável no acompanhamento do curso e tratamento da doença. Calcule a importância da importação para testes de avaliação clínica para doença que se tornou possível quantificar o progresso da incapacidade na EM e comparar dados de estudos populacionais sob uma base útil.[32]

Em 1955, foi desenvolvido um sistema de pontuação para incapacidade (DSS) que, posteriormente foi expandido, sendo então denominado Escala de Status de Incapacidade Expandida (EDSS) ou Escala de Incapacidade Funcional Expandida.[32]

This scale is based on a standard neurological assessment of 8 functional systems (Annex 2). Their scores range from 0 to 10 with the highest scores reflecting worse disability and difficulty walking. EDSS focuses on ambulation measurements, including the ability to travel multiple distances with or without assistance.[27]

3.3.2. Nitric Oxide (ON) Evaluation

The evaluation of NO is a complex science because it is involved in several physiological processes, including blood pressure regulation, immune response and neural communication. Therefore, its accurate detection and quantification are critical for understanding health and disease.[33]

O nitrito é uma molécula homeostática central de biologia ON e é um importante sinalizador de ON sintase.[33]

A concentração e quantificação de nitritos será determinada nas quantidades de sangue de acordo com o método Griess.

3.3.3. IL 10 evaluation

Interleukin 10 (IL-10) is a widely studied cytokine known primarily for regulating and suppressing expression of proinflammatory cytokines such as TNF-α, IL-1β and IL-6, improving Th1 and CD8 lymphocyte responses. . It also inhibits the generation of reactive oxygen species. (ROS).[34.35.36]

IL-10 acts predominantly in an anti-inflammatory manner and modulates the inflammatory response and appears to be even more evident in chronic inflammatory conditions, especially in those in which TNF-α appears to act significantly as for example in MS.[36]

The models of autoimmune diseases in IL-10 deficient mice helped to elucidate the role of this cytokine in T lymphocyte homeostasis, as they were more prone to inflammatory diseases, and when these animals were treated coding for the human IL-10 gene, obtained beneficial effects. Thus, this cytokine has been gaining a prominent role and a therapeutic possibility.[36]

Recent studies in animal models with inflammatory lung diseases have shown that after treatment with photobiomodulation the levels of proinflammatory cytokines IL-6 and TNF-alpha were reduced and IL-10 concentrations were high, concluding that photobiomodulation may stimulate this. cytokine and thereby improve inflammatory processes.[37]

IL-10 concentration and quantitation will be determined in blood samples according to the ELISA method.

3.4. Photobiomodulation Protocol

Regarding the photobiomodulation protocols, the articles of interest were identified by conducting a search by title and abstract. Articles whose theme was not relevant to the research were excluded (Table 1). Secondly, the selected articles were analyzed, after which an initial protocol was created.

Table 1 - Summary of parameters used in the literature

**Article**	**Power**	**time**	**Radiant Exposure**	**Total energy**	**Wavelength**	**Study Type**
Authors, year	***mW***	**seg**	**J/cm²**	**J**	**NM**	***In vivo, vitro, clinical trial***
Muili, 2013	2100	180	5	375	670	vivo
Muili, 2012	2100	180	5	375	670	vitro
Gonçalves, 2015	30	20	10	0,6 J	660	vivo

Gonsalves, 2015	70 W	20	3		904	vivo
Kubsik, 2016	50	30		3	650	Clinical trial
song	64.6				632.8	vitro
Ailioaie, 2014					635,	Clinical trial

Spinal cord irradiation will be applied transcutaneously to the segments corresponding to the lumbosacral (T12-S5) and cervico-thoracic (C5-T1-2) nerve roots, will be irradiated 20 points for 30 seconds and the total treatment time was 10 minutes. The group that will receive the irradiation with laser therapy device, model EC, DMC brand in the sublingual region, will use a disposable plastic material covering the application pen, for hygiene reasons, the total treatment time will be 10 minutes. Intravascular laser irradiation (ILIB) will be applied to the radial artery region with a specific wristband of the DMC Model Therapy EC laser diode apparatus for 10 minutes (TABLE 1). The participants of the placebos groups will be positioned in the same way, but will not receive light emission.

The treatment will be performed twice a week for a total of 24 consecutive weeks and after 3 months of treatment the patients will be reassessed of all the complementary exams that were requested, cytokines, nitric oxide and EDSS.

Table 1 - Parameters

**Parameter**	**Unidade**	**Spinal Cord**	**Sublingual**	**ILIB**
Center wavelength	nm	808	808	660

3.5 Statistical analysis

The data will be tabulated and processed in the program SPSS 20.0 for Win and the descriptive statistics will be performed. To evaluate the association of categorical variables, the chi-square and Fisher's exact test will be used, Student's t-test will be used and for the analysis of the correlation between continuous variables, the Pearson's correlation test will be applied. A significance level of 95% ($p < 0.05$) will be considered.

3.6. Sample Calculation

The sample size was calculated to ensure a 95% test power grater, so with 34 subjects and an effect size of 0.8, the test power is 0.9566, keeping the significant level at $\alpha = 0.05$ (Figure 1).

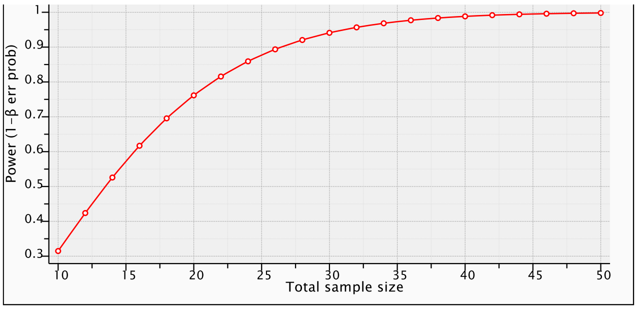

Figure 1.

References

1. Ransohoff RM, et al. Multiple sclerosis—a quiet revolution. Rev Neurol. 2015 Mar;11;3:134–142
2. Albarracín JDF, Segura OME. Esclerosis múltiple en pacientes pediátricos: fisiopatología, diagnóstico y manejo. Rev. Med UNABI Vol. 2012 Mar14(3):167-179.
3. Kubsik A, et al. Application of laser radiation and magnetostimulation in therapy of patients with multiple sclerosis.NeuroRehabilitation. 2016 183–190
4. Deckx N, et al. 2 Weeks of Combined Endurance and Resistance Training Reduces Innate Markers of Inflammation in a Randomized Controlled Clinical Trial in Patients with Multiple Sclerosis. Mediators of InflammationVolume 2016, Article ID 6789276, 13 pageshttp://dx.doi.org/10.1155/2016/6789276
5. Paloczi J, Varga ZV, Hasko G, et al. Neuroprotection in oxidative stress-related neurodegenerative diseases: role of endocannabinoid system modulation. Antioxid Redox Signal. 2017 Jul;18:2-95
6. Morel A, Bijak M, Niwald M, et al. Markers of oxidative/nitrative damage of plasma proteins correlated with EDSS and BDI scores in patients with secondary progressive multiple sclerosis. Redox Report. 2017 May: https://doi.org/10.1080/13510002.2017.1325571.
7. Matejčíkováa Z, Mareš J, Sládková V, et al. Cerebrospinal fluid and serum levels of interleukin-8 in patients with multiple sclerosis and its correlation with Q-albumin. Multiple Sclerosis and Related Disorders 2017 Mar;14 12–15

8. Murphy AC, Lalor SJ, Lync, et al. Infiltration of Th1 and Th17 cells and activation of microglia in the CNS during the course of experimental autoimmune encephalomyelitis. Brain, Behavior, and Immunity. 2010 Feb;24:641–651

9. Pilli D, Zou A, Tea F, et al. Expanding Role of T Cells in Human Autoimmune Diseases of the Central Nervous System. Frontiers in Immunology. 2017;8:1-16.

10. Lisak, RP, Nedelkoska L, Benjamins JA, et al. B cells from patients with multiple sclerosis induce cell death via apoptosis in neurons in vitro. Journal of Neuroimmunology. 2017 Feb; doi: 10.1016/j.jneuroim.2017.05.004.

11. Muili KA, Gopalakrishnan S, Meyer SL, Eells JT, Lyons JA. Amelioration of Experimental Autoimmune Encephalomyelitis in C57BL/6 Mice by Photobiomodulation Induced by 670 nm Light. PLoS ONE. 2012 Jan;7:1-9.

12. Voigt D, Scheidt U, Derfuss T, et al. Expression of the Antioxidative Enzyme Peroxiredoxin 2 in Multiple Sclerosis Lesions in Relation to Inflammatio. Int. J. Mol. Sci. 2017, 18, 760; doi:10.3390/ijms18040760

13. Ghasemi M, Fatemi A. Pathologic role of glial nitric oxide in adult and pediatricneuroinflammatory diseases. Neuroscience and Biobehavioral Reviews 2014;5:168–182

14. Calabrese V, Calabrese C, Rizzarelli E, et al. Nitric oxide in the central nervous system: neuroprotection versus neurotoxicity. Nature Publishing Group. 2007 oct;8: 766 – 775.

15. Hofling DB, Chavantes MC, Acencio MMP, et al. Effects of Low-Level Laser Therapy on the Serum TGF-b1 Concentrations in Individuals with

Autoimmune Thyroiditis. Photomedicine and Laser Surgery. 2014; 32:8 444–449.

16. Nakamura, T., Cho, D.H., Lipton, S.A. Redox regulation of protein misfolding,mitochondrial dysfunction, synaptic damage, and cell death in neurodegenera-tive diseases. Exp. Neurol.2012; 238:12–21.
17. Gonçalves DG, et al. Low-level laser therapy ameliorates disease progression in a mouse model of multiple sclerosis. Autoimmunity. 2016;49:2:132-42
18. Peterson JW, Bö L, Mörk S, Chang A, Trapp BD. Transected Neurites, Apoptotic Neurons, and Reduced Inflammation in Cortical Multiple Sclerosis Lesions. Ann Neurol. 2001 Sep;50(3):389-400
19. Costello F. Vision Disturbances in Multiple Sclerosis. Semin Neurol. 2016 Apr;36;2:185-95.
20. Hempel, S, et al. A systematic review of modifiable risk factors in the progression of multiple sclerosis. Mult Scler. 2017 Apr;23;4:525-533
21. Alvarenga FH, Sacramento PM, Ferreira TB, et al. Combined exercise training reduces fatigue and modulates the cytokine profile of Tcells from multiple sclerosis patients in response to neuromediators. J Neuroimmunol. 2016 Apr 15;293:91-9
22. B Huppke et.al. Clinical presentation of pediatric multiple sclerosis before puberty. European Journal of Neurology 2014, 21: 441–446
23. Rubin JP, Kuntz NL. Diagnostic Criteria for Pediatric Multiple Sclerosis.Curr Neurol Neurosci Rep. 2013; 13:354
24. Silva LR. Challenges in Diagnosis and Treatment of Pediatric Multiple Sclerosis. Acta Med Port 2016 Jul-Aug;29(7-8):419-420

25. Albarracín JDF, Segura OME; Esclerosis múltiple en pacientes pediátricos: fisiopatología, diagnóstico y manejo. Rev. Med UNABI Vol. 14(3):167-179, Março 2012

26. B Huppke et.al. Clinical presentation of pediatric multiple sclerosis before puberty. European Journal of Neurology 2014, 21: 441–446

27. Expanded Disability Status Scale (EDSS).Data from Kurtzke JF. Neurology. 1983;33:1444-1452.

28. Filho H A, et al**.** Combined exercise training reduces fatigue and modulates the cytokine profile of T cells from multiple sclerosis patients in response to neuromediators. J Neuroimmunol. 2016 Apr 15;293:91-9.

29. Learmonth YC, Adamson BC, Balto JM et al. Investigating the needs and wants of healthcare providers for promoting exercise in persons with multiple sclerosis: a qualitative study. Disabil Rehabil. 2017 May; 19:1-9

30. Muili KA, Gopalakrishnan S, Meyer SL, Eells JT, Lyons J-A Photobiomodulation Induced by 670 nm Light Ameliorates MOG35 Induce EAE in Famale C57BL/6 Mice: A Role for Remediation of Nitrosative Stress. PLoS One. 2013 Jun 28;8(6):e67358

31. Ailioaie LM, Litscher G, WeberM, Litscher D, Chiran DA. Innovations and Challenges by Applying Sublingual Laser Blood Irradiation in Juvenile Idiopathic Arthritis. Int J of Photoenergy . 2014 may http://dx.doi.org/10.1155/2014/130417

32. Kurtzke, MD. Rating neurologic impairment in multiple sclerosis: An expanded disability status scale (EDSS). NEUROLOGY 1983 Feb 33:1444-52

33. Bryan NS, Grisham MB. Methods to Detect Nitric Oxide and its Metabolites in Biological Samples. Free Radic Biol Med. 2007 Sep 1; 43(5): 645–657.
34. Kevin N. Couper, Daniel G. Blount and Eleanor M. Riley. IL-10: The Master Regulator of Immunity to Infection J Immunol May 1, 2008, 180 (9) 5771-5777; DOI: https://doi.org/10.4049/jimmunol.180.9.5771
35. Rojas JM, Avia M, MartínV, Sevilla N. IL-10: A Multifunctional Cytokine in Viral Infections. J of Immunol Res. 2017 http://dx.doi.org/10.1155/2017/6104054
36. Junior BLM , Delascio RL, Seelaender MCL, Lopes AC. Anti-inflammatory Effect of Physical Training in Heart Failure: Role of TNF-α and IL-10. Arq Bras Cardiol 2009 Out 93(6) : 692-700
37. Macedo RS, Leal MP, Braga TT. Photobiomodulation Therapy Decreases Oxidative Stress in the Lung Tissue after Formaldehyde Exposure: Role of Oxidant/Antioxidant Enzymes. Mediators of Inflammation. 2016 May; http://dx.doi.org/10.1155/2016/9303126.
