# Supplementary material for: Effects of photobiomodulation on interleukin-10 and nitrites in individuals with relapsing-remitting multiple sclerosis – Randomized clinical trial
Source: PLoS One. 2020 Apr 7;15(4):e0230551. doi: 10.1371/journal.pone.0230551 (PMC7138327; doi:10.1371/journal.pone.0230551)
Supplement: S9 File — (PDF) [file pone.0230551.s009.pdf]

UNINOVE

Universidade Nove de Julho

A EFETIVIDADE DA FOTOBIOMODULAÇÃO EM INDIVIDUOS COM ESCLEROSE MÚLTIPLA APÓS A APLICAÇÃO NA MEDULA ESPINAL E SUBLINGUAL – ENSAIO CLÍNICO, CONTROLADO, ALEATORIZADO.

São Paulo

2017

1. Enunciado do Problema

1.1. Esclerose Múltipla

A esclerose múltipla (EM) é uma desordem inflamatória desmielinizante e neurodegenerativa do sistema nervoso central (SNC) que se caracteriza por destruir de forma seletiva a bainha de mielina[1-2]. A sua etiologia é multifatorial, complexa e não é totalmente compreendida, porém, acredita-se que formação de lesões desmielinizantes é devido aos processos autoimunes, e também um resultado de fatores ambientais e genéticos[3-4].

Embora os mecanismos precisos que desencadeiam a doença não tenham sido totalmente elucidados, sabe-se que ocorre uma desregulação das células Th1, Th17 e CD4, elas são ativadas na periferia e migram principalmente através da barreira hematoencefálica (BHE) invadindo o SNC e interagem com células apresentadoras de antígenos (CAA), a saber, os astrócitos, micróglia, macrófagos e células dendríticas (DC), induzindo a produção de citocinas pró-inflamatórias, tais como,, interleucina(IL) 17 e fator de necrose tumoral alfa (TNF- α)[5,6,7,8,9].

Os linfócitos B atuam como células CAA específicos para as células T, e quando ativadas também penetram através de BHE, e produzem anticorpos específicos para os antígenos de mielina, com isso a mielina se torna alvo para células imunes, que a reconhece como um antígeno estranho e a destrói [6,7,10].

Além disso, algumas evidências relatam um papel importante do estresse oxidativo na patogênese da EM e à sua contribuição para a inflamação, lesão de oligodendrócitos, alterações na transmissão

sináptica, degeneração axonal e morte neuronal, sugerindo que estresse oxidativo contribui para a neurodegeneração[5,11].

O estresse oxidativo acontece devido ao acúmulo de radicais livres, ou seja, espécies reativas de oxigênio (ROS) e espécies de reativas de nitrogênio (RNS)[5,12]. Os recentes estudos relatam que existe uma relação entre neurônios e células imunes, que mantém e garante a homeostase do SNC e o desequilíbrio entre a produção de radicais livres e a capacidade antioxidante das células é responsável pelo dano neuronal[5,6].

Em condições normais a ROS e RNS alteram as proteínas e danificam o DNA / RNA, no entanto, as células do SNC são capazes de se protegerem contra ROS e RNS, inativando essas moléculas altamente reativas e reparando danos emergentes. Esta capacidade depende criticamente da expressão de moléculas antioxidativas. O SNC é particularmente sensível à oxidação devido ao seu alto consumo de oxigênio e ao nível relativamente baixo de antioxidantes endógenos [5,6].

O óxido Nítrico (ON) é uma ERN, e possui um papel importante na patogênese da EM contribuindo com a inflamação, lesões dos oligodendrócitos, alteração nas sinapses, degeneração axonal e morte neural[5]. Em baixas concentrações, desempenha papel fisiológico nas células, incluindo neurotransmissão, regulação do tom do vaso sanguíneo e resposta imune, e em concentrações mais elevadas, está implicado na patogênese de várias doenças neurológicas como as doenças neurodegenerativas, desmielinizantes e doenças neuroinflamatórias[13,14].

O ON é enzimaticamente produzido a partir do aminoácido l-arginina através das atividades de síntese das enzimas de ON (NOS). Após a

degradação destas enzimas, são identificados três subtipos de NOS, a saber, NOS Neuronal (nNOS ou NOS1), NOS endotelial (eNOS ou NOS3) e NOS induzido (iNOS ou NOS2. Este último tipo indica que a expressão da enzima é induzida por estímulos inflamatórios agudos. iNOS é expresso em vários tipos de células, especialmente as células gliais, principlamente, astrócito e micrógliaA micróglia expressa somente o iNOS em condições patológicas, tais como, isquemia, trauma, danos neurotóxicos e inflamatórias incluindo a EM[15,13, 16].

As micróglias são residentes no SNC e fisiologicamente realizam uma variedade de tarefas, que incluem manutenção celular, liberação de fatores tróficos e anti-inflamatórios que facilitam a migração de células-tronco para o local de inflamação, e também possuem capacidades semelhantes aos macrófagos, incluindo fagocitose e produção de citocina inflamatória[15]. Porém quando ativada e desencadeia respostas pró-inflamatórias e altera o equilíbrio oxidante / antioxidante, levando a perda neuronal[5,15,13].

A micróglia e os astrócitos representam uma fonte importante de moléculas neuro toxicas, que são os medicadores inflamatórios, espécie reativa de oxigênio (ERO) e nitrogênio (ERN), os mesmos responsáveis pela perda progressiva da estrutura e função da célula neuronais[5].

O resultado da inflamação e o estresse oxidativo é o dano da mielina e oligodendrócitos, causando uma multiplicidade de sintomas[, 7,18].

Os sintomas iniciais são heterogêneos com progressão variável ao longo do tempo, incluindo alterações sensoriais, fadiga incapacidade física e / ou mental, distúrbios do equilíbrio, espasticidade, fraqueza

muscular, incontinência urinária, alterações cognitivas, dor neuropática, distúrbios visuais[3,4,17,19,20,21].

A doença se manifesta de várias formas, tais como: Remitente Recorrente (RR), que se caracteriza por recidivas súbitas pontuadas por remissões de curto ou longo prazo; Secundariamente Progressiva (SP), que tem um curso progressivo resultando em debilitação grave e irreversível e Primariamente Progressiva (PP), que é um tipo progressivo de EM sem recorrência inicial e período de remissão[17,19,22]. Em geral, as recidivas são devido a inflamação focal aguda e a progressão da doença é causada por neurodegeneração crônica[7].

O diagnóstico de EM em adultos e crianças requer episódios recorrentes de desmielinização do SNC, achados no exame de imagem e líquido cefalorraquidiano (LCR), nível elevado no plasma sanguíneo de proteínas do dano oxidativo / nitrativo, o que é importante para fornecer informações sobre o processo inflamatório, além da apresentação clínica[6,23].

A EM apresentam-se tipicamente entre as idades de 20-40 anos, porém pode manifestar-se durante a infância ou adolescência. A incidência global de EM na infância é desconhecida e poucos estudos epidemiológicos exibem resultados, estima-se que 3% a 10% de todos os pacientes com EM terão início antes de 18 anos de idade[1,24,25].

O prognóstico é imprevisível em relação à incapacidade que ocorre devido às sequelas, normalmente associadas a comprometimento progressivo da locomoção[21]. Em relação à criança, apesar de um curso

inflamatório mais precoce, a recuperação inicial é melhor e a progressão da doença é mais lenta em crianças do que em adultos[26].

Escala de Status de Expansão Expandida (EDSS) é um método para quantificar e monitorar a incapacidade de indivíduos com EM. Escores mais elevados no EDSS correspondem a maior gravidade da doença clinicamente[3,6,27].

O tratamento pode ser realizado através fármacos, e a reabilitação que tem mostrado melhorias na caminhada, cognição, fadiga, depressão, QV, participação na tarefa, melhora a força muscular, o desempenho cardiovascular, funcionamento mental, equilíbrio, fadiga e a qualidade de vida[3,4,28,29].

Estudos in vitro e in vivo têm demonstrado a eficácia da fotobiomodulação (FBM) em doenças inflamatórias, incluindo doenças desminielizantes como a EM[6,16]. A FBM pode modular um amplo espectro de processos celulares, incluindo: Evitar a morte de células e tecidos, estimulação de cicatrização e reparação de lesões, redução da dor, edema e inflamação, proliferação até a apoptose[15,17].

Estudos mostraram que a FBM provoca uma sequência de alterações ao nível celular, e o resultado é, entre outros, a regeneração de células, incluindo a estimulação do crescimento de células de Schwann, diminuição da espasticidade e melhora funcional, além reduzir eficientemente os níveis de ON[3,15,17]. A fotobiomodulação no modelo experimental de encefalopatia autoimune (EAE) teve como resultado uma regulação positiva da IL10, que é uma citocina anti-inflamatória mostrando

que a fotobiomodulação pode oferecer neuro-proteção no modelo EAE[11,30].

Recentemente, a FBM foi utilizada para o controle da inflamação, evitar danos articulares ou perda de função, e diminuir a dor e lesão sistêmica na Artrite Reumatoide Juvenil (ARJ), este estudo teve como objetivo introduzir uma nova técnica e investigar os efeitos da FBM sublingual em pacientes com ARJ, com diferentes comprimentos de onda. A circulação sanguínea é muito abundante na língua dorsal das crianças, por isso a irradiação com laser desta área pode trazer grandes benefícios, sendo não invasiva e indolor. Os resultados mostraram a redução da dor, aumentou a qualidade de vida[31].

1.2. Justificativa

Os tratamentos para a esclerose múltipla (EM) destinam-se a resposta imune e a progressão lenta da doença. Sabe-se que na EM as citocinas inflamatórias de Th1 estão presentes e que existe uma alta concentração de Oxido Nítrico, e os estudos in vivo e in vitro demostraram que a fotobiomodulação pode modular os níveis de óxido nítrico. Porém a literatura se mostrou escassa de estudos sobre o assunto principalmente, ensaios clínicos controlados e não há estudos que mostrem o efeito da fotobiomodulação sublingual, e foi encontrado apenas 1 estudo realizado em humanos onde o laser foi aplicado na região da medula espinal.[3]
Sendo assim a fotobiomodulação sublingual e medular podem ser uma terapia da doença de forma não farmacológica promissora para o tratamento de condições autoimunes, tais como a EM.

2. Objetivo

Avaliar a eficácia da fotobiomodulação nas regiões medular, sublingual e artéria radial em indivíduos com Esclerose Múltipla.

2.1 Objetivos secundários

- Avaliar a funcionalidade através o EDSS em indivíduos com Esclerose Múltipla;
- Avaliar a expressão da IL-10, TNF- α e ON
- Comparar a fotobiomodulação na região sublingual e medular em pacientes com esclerose múltipla
- Avaliar se a fotobiomodulação possui efeito sistêmico

3. Materiais e métodos

3.1. Delineamento do estudo

Este estudo será um ensaio clínico, controlado e aleatorizado que será desenvolvido segundo o fluxograma apresentado (FIGURA 1). Seguirá as normas regulamentadoras (resolução número 466/2012) de pesquisa em seres humanos com submissão e aprovação do Comitê de Ética em pesquisa da Universidade Nove de Julho. Os participantes ou responsáveis assinarão o Termo de Consentimento Livre e Esclarecido (TCLE) e Termo de assentimento (TA) para autorização da participação na pesquisa (ANEXO1).

O processo de recrutamento será no ambulatório integrado de Saúde da Universidade Nove de Julho (UNINOVE), no campus memorial e vila maria. Os pacientes que possuírem o diagnóstico de EM, serão triados por telefone e se atenderem aos critérios de inclusão e dão consentimento informado por escrito

devem ser randomizados. A randomização será estratificada por centro com tabelas usando alocação de bloco para fornecer distribuição de tratamento em proporções iguais. Os participantes e o avaliador não saberão em quais grupos ficarão.

Será coletado amostras de 10 ml de sangue por um enfermeiro onde será avaliado o estresse oxidativo (nitrito/nitrato), mediadores inflamatórios (TNF- α) e investigar o efeito anti-inflamatório da fotobiomodulação (IL-10) . A avaliação dessas citocinas será através do teste ELISA e reação de griess. E também será avaliado as incapacidades físicas dos participantes através da Escala de Status de Expansão Expandida (EDSS), que será aplicada em 20 minutos.

Após a avaliação os participantes serão divididos em 6 grupos: Grupo 1 receberá tratamento placebo com fotobiomodulação sublingual, grupo 2 receberá tratamento com fotobiomodulação na região sublingual, Grupo 3 receberá placebo com fotobiomodulação na região medular, Grupo 4 receberá o tratamento com fotobiomodulação na região medular. grupo 5 receberá tratamento com fotobiomodulação na região da artéria radial, grupo 6 receberá placebo com fotobiomodulação na região artéria radial. É importante ressaltar que os participantes de todos os grupos não serão privados de nenhuma medicação para o tratamento de sua condição de base. Se houver melhora nos grupos de tratamento com a fotobiomodulação, todos os participantes que forem sorteados para ficarem no grupo do tratamento fictício, receberão o tratamento real com a fotobiomodulação, imediatamente após o término do estudo, para que não fique em desvantagem em relação ao outro grupo.

3.2. Casuística

Serão inclusos na pesquisa, indivíduos com diagnóstico de Esclerose Múltipla, EDSS até 6, com idade de 18 a 60 anos, e deverão estar realizando tratamento farmacológico. Indivíduos que apresentarem outras doenças autoimunes, neoplasias, insuficiência cardíaca, insuficiência respiratória, insuficiência renal, insuficiência hepática, síndrome da imunodeficiência adquirida e apresentarem surtos da doença serão excluídos da pesquisa.

3.3. Avaliações

3.3.1. Avaliação funcional - Expanded Disability Status Scale (EDSS)

A EM é considerada uma das doenças mais incapacitantes dentre as relacionadas ao SNC e a mensuração desta incapacidade funcional se faz indispensável no acompanhamento do curso e tratamento da doença. Devido a esta importabncia foram desenvolvidas escalas de avaliação clínica para a doença que tornou-se possível quantificar a progressão da incapacidade na EM e comparar dados de estudos populacionais sob uma base significativa[32].

Em 1955, foi desenvolvido um sistema de pontuação para incapacidade (DSS) que, posteriormente foi expandido, sendo então denominado Expanded Disability Status Scale (EDSS) ou Escala de Incapacidade Funcional Expandida[32].

Essa escala é baseada em uma avaliação neurológica padrão de 8 sistemas funcionais, (Anexo 2). Sua pontuação varia de 0 a 10 sendo que os maiores escores refletem pior incapacidade e dificuldade de caminhada. O EDSS centra-se em medidas de deambulação, incluindo a capacidade de percorrer várias distâncias com ou sem ajuda[27].

3.3.2. Avaliação do Óxido Nítrico (ON)

A avaliação do ON é uma ciência complexa, pois está envolvido em diversos processos fisiológicos, incluindo regulação da pressão sanguínea, resposta imune e comunicação neural. Portanto, sua detecção e quantificação precisas são críticas para a compreensão da saúde e da doença[33].

O nitrito é uma molécula homeostática central na biologia ON e é um importante sinalizador do ON sintase[33].

A concentração e quantificação de nitritos será determinada nas amostras de sangue de acordo com o método de Griess.

3.3.3. Avaliação da IL 10

A Interleucina 10 (IL-10) é uma citocina muito estudada e conhecida principalmente por regular e suprimir a expressão de citocinas pró-inflamatórias, tais como TNF-α, IL-1β e IL-6, melhorando as respostas dos linfócitos Th1 e CD8. Também inibe a geração de espécies reativas do oxigênio (ERO)[34,35,36].

A IL-10 atua predominantemente de maneira anti-inflamatória e modula a resposta inflamatória e parece ser ainda mais evidente em

condições inflamatórias crônicas, principalmente naquelas em que o TNF-α parece atuar de maneira significativa como por exemplo na EM[36].

Os modelos de doenças autoimunes em camundongos deficientes em IL-10 ajudaram a elucidar o papel desta citocina na homeostase dos linfócitos T, pois apresentaram maior pré-disposição a doenças inflamatórias, e quando esses animais foram tratados codificador do gene para IL-10 humano, obteve efeitos benéficos. Dessa forma, essa citocina vem ganhando um papel de destaque, e uma possibilidade terapêutica[36].

Estudos recentes em modelos animais com doenças inflamatórias pulmonares, mostraram que após o tratamento com fotobiomodulação os níveis de citocinas pró-inflamatórias IL-6 e TNF-alfa foram reduzidos e as concentrações de IL-10 foram elevadas, concluindo que a fotobiomodulação pode estimular essa citocina e com isso melhorar processos inflamatórios[37].

A concentração e quantificação da IL-10 será determinada nas amostras de sangue de acordo com o método ELISA.

3.4. Protocolo Fotobiomodulação

Quanto aos protocolos da fotobiomodulação, os artigos de interesse foram identificados a partir da realização de uma pesquisa pelo título e resumo. Os artigos cujo tema não era pertinente à pesquisa foram excluídos (quadro1). Em um segundo momento, os artigos selecionados foram analisados, após isso foi criado um protocolo inicial.

Quadro 1 - Resumo dos parâmetros utilizados na literatura

Artigo	Potencia	Tempo Total	Exposição radiante	Energia Total	Comprimento da onda	Tipo de estudo
Autores, ano	***mW***	**seg**	**J/cm²**	**J**	**NM**	***In vivo, vitro, Ensaio clínico***
Muili, 2013	2100	180	5	375	670	vivo
Muili, 2012	2100	180	5	375	670	vitro
Gonçalves, 2015	30	20	10	0,6 J	660	vivo
Gonsalves, 2015	70 W	20	3 fluência		904	vivo
Kubsik, 2016	50	30		3	650	Ensaio Clínico
song	64.6				632.8	vitro
Ailioaie, 2014					635,	Ensaio clínico,

A irradiação medular será aplicada por via transcutânea nos segmentos correspondentes as raízes nervosas do plexo lombo-sacral (T12-S5) e cervico-toracico (C5-T1-2), será irradiado 20 pontos por 30 segundos e o tempo total do tratamento foi de 10 minutos. O grupo que receberá a irradiação com aparelho de diodo laser, modelo therapy EC, da marca DMC na região sublingual, será utilizado um material plástico descartável cobrindo a caneta de aplicação, por questões de higiene, o tempo total do tratamento será de 10 minutos. A irradiação com laser intravascular (ILIB) será aplicada na região da artéria radial com pulseira específica do aparelho de diodo laser, Modelo Therapy EC, da marca DMC por 10 minutos (TABELA 1). Os participantes dos grupos placebos serão posicionados da mesma maneira, porém não receberá emissão da luz.

O tratamento, será realizado 2 vezes por semana totalizando 24 semanas consecutivas e após 3 meses de tratamento os pacientes serão submetidos a

reavaliação de todos os exames complementares que foram solicitados, citocinas, óxido nítrico e EDSS.

Tabela 1 - Parâmetros do Laser

**Parameter**	**Unidade**	**Spinal Cord**	**Sublingual**	**ILIB**
Center wavelength	nm	808	808	660

3.5. Analise estatÍstica

Os dados serão tabulados e tratados no programa SPSS 20.0 for Win e será realizada a estatística descritiva. Para avaliação da associação das variáveis categóricas será utilizado o teste Qui-quadrado e Exato de Fisher, será utilizado teste t-Student e para análise da correlação entre as variáveis contínuas será aplicado o teste de correlação de Pearson. Será considerado um nível de significância de 95% ($p<0,05$).

3.6. Cálculo amostral

O tamanho da amostra foi calculado para assegurar um ralador de potência de teste de 95%, portanto, com 34 indivíduos e um tamanho de efeito de 0,8, o poder de teste é de 0,9566, mantendo o nível significativo em α = 0,05(figura 1).

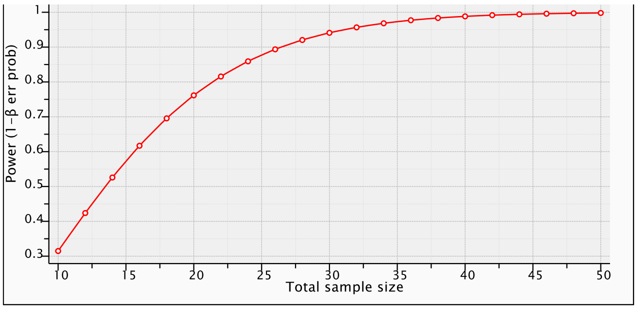

Figura 1

4. Cronograma

Atividades	Semestres			
	1º	2º	3º	4º
Revisão da Literatura	X			
Escrever material e métodos	X			
Encaminhar para COeP		X		
Triagem dos voluntários		X		
1ª Qualificação			X	
Execução da metodologia		X	X	X
Análise dos dados				X
Escrever Resultados			X	X
Escrever Discussão e Conclusão				X
Defesa da tese				X
Encaminhar artigo para publicação				X

5. Referencias

1. Ransohoff RM, et al. Multiple sclerosis—a quiet revolution. Rev Neurol. 2015 Mar;11;3:134–142
2. Albarracín JDF, Segura OME. Esclerosis múltiple en pacientes pediátricos: fisiopatología, diagnóstico y manejo. Rev. Med UNABI Vol. 2012 Mar14(3):167-179.
3. Kubsik A, et al. Application of laser radiation and magnetostimulation in therapy of patients with multiple sclerosis.NeuroRehabilitation. 2016 183–190
4. Deckx N, et al. 2 Weeks of Combined Endurance and Resistance Training Reduces Innate Markers of Inflammation in a Randomized Controlled Clinical Trial in Patients with Multiple Sclerosis. Mediators of InflammationVolume 2016, Article ID 6789276, 13 pageshttp://dx.doi.org/10.1155/2016/6789276
5. Paloczi J, Varga ZV, Hasko G, et al. Neuroprotection in oxidative stress-related neurodegenerative diseases: role of endocannabinoid system modulation. Antioxid Redox Signal. 2017 Jul;18:2-95
6. Morel A, Bijak M, Niwald M, et al. Markers of oxidative/nitrative damage of plasma proteins correlated with EDSS and BDI scores in patients with secondary progressive multiple sclerosis. Redox Report. 2017 May: https://doi.org/10.1080/13510002.2017.1325571.
7. Matejčíkováa Z, Mareš J, Sládková V, et al. Cerebrospinal fluid and serum levels of interleukin-8 in patients with multiple sclerosis and its correlation with Q-albumin. Multiple Sclerosis and Related Disorders 2017 Mar;14 12–15

8. Murphy AC, Lalor SJ, Lync, et al. Infiltration of Th1 and Th17 cells and activation of microglia in the CNS during the course of experimental autoimmune encephalomyelitis. Brain, Behavior, and Immunity. 2010 Feb;24:641–651

9. Pilli D, Zou A, Tea F, et al. Expanding Role of T Cells in Human Autoimmune Diseases of the Central Nervous System. Frontiers in Immunology. 2017;8:1-16.

10. Lisak, RP, Nedelkoska L, Benjamins JA, et al. B cells from patients with multiple sclerosis induce cell death via apoptosis in neurons in vitro. Journal of Neuroimmunology. 2017 Feb; doi: 10.1016/j.jneuroim.2017.05.004.

11. Muili KA, Gopalakrishnan S, Meyer SL, Eells JT, Lyons JA. Amelioration of Experimental Autoimmune Encephalomyelitis in C57BL/6 Mice by Photobiomodulation Induced by 670 nm Light. PLoS ONE. 2012 Jan;7:1-9.

12. Voigt D, Scheidt U, Derfuss T, et al. Expression of the Antioxidative Enzyme Peroxiredoxin 2 in Multiple Sclerosis Lesions in Relation to Inflammatio. Int. J. Mol. Sci. 2017, 18, 760; doi:10.3390/ijms18040760

13. Ghasemi M, Fatemi A. Pathologic role of glial nitric oxide in adult and pediatricneuroinflammatory diseases. Neuroscience and Biobehavioral Reviews 2014;5:168–182

14. Calabrese V, Calabrese C, Rizzarelli E, et al. Nitric oxide in the central nervous system: neuroprotection versus neurotoxicity. Nature Publishing Group. 2007 oct;8: 766 – 775.

15. Hofling DB, Chavantes MC, Acencio MMP, et al. Effects of Low-Level Laser Therapy on the Serum TGF-b1 Concentrations in Individuals with

Autoimmune Thyroiditis. Photomedicine and Laser Surgery. 2014; 32:8 444–449.

16. Nakamura, T., Cho, D.H., Lipton, S.A. Redox regulation of protein misfolding,mitochondrial dysfunction, synaptic damage, and cell death in neurodegenera-tive diseases. Exp. Neurol.2012; 238:12–21.
17. Gonçalves DG, et al. Low-level laser therapy ameliorates disease progression in a mouse model of multiple sclerosis. Autoimmunity. 2016;49:2:132-42
18. Peterson JW, Bö L, Mörk S, Chang A, Trapp BD. Transected Neurites, Apoptotic Neurons, and Reduced Inflammation in Cortical Multiple Sclerosis Lesions. Ann Neurol. 2001 Sep;50(3):389-400
19. Costello F. Vision Disturbances in Multiple Sclerosis. Semin Neurol. 2016 Apr;36;2:185-95.
20. Hempel, S, et al. A systematic review of modifiable risk factors in the progression of multiple sclerosis. Mult Scler. 2017 Apr;23;4:525-533
21. Alvarenga FH, Sacramento PM, Ferreira TB, et al. Combined exercise training reduces fatigue and modulates the cytokine profile of Tcells from multiple sclerosis patients in response to neuromediators. J Neuroimmunol. 2016 Apr 15;293:91-9
22. B Huppke et.al. Clinical presentation of pediatric multiple sclerosis before puberty. European Journal of Neurology 2014, 21: 441–446
23. Rubin JP, Kuntz NL. Diagnostic Criteria for Pediatric Multiple Sclerosis.Curr Neurol Neurosci Rep. 2013; 13:354
24. Silva LR. Challenges in Diagnosis and Treatment of Pediatric Multiple Sclerosis. Acta Med Port 2016 Jul-Aug;29(7-8):419-420

25. Albarracín JDF, Segura OME; Esclerosis múltiple en pacientes pediátricos: fisiopatología, diagnóstico y manejo. Rev. Med UNABI Vol. 14(3):167-179, Março 2012

26. B Huppke et.al. Clinical presentation of pediatric multiple sclerosis before puberty. European Journal of Neurology 2014, 21: 441–446

27. Expanded Disability Status Scale (EDSS).Data from Kurtzke JF. Neurology. 1983;33:1444-1452.

28. Filho H A, et al**.** Combined exercise training reduces fatigue and modulates the cytokine profile of T cells from multiple sclerosis patients in response to neuromediators. J Neuroimmunol. 2016 Apr 15;293:91-9.

29. Learmonth YC, Adamson BC, Balto JM et al. Investigating the needs and wants of healthcare providers for promoting exercise in persons with multiple sclerosis: a qualitative study. Disabil Rehabil. 2017 May; 19:1-9

30. Muili KA, Gopalakrishnan S, Meyer SL, Eells JT, Lyons J-A Photobiomodulation Induced by 670 nm Light Ameliorates MOG35 Induce EAE in Famale C57BL/6 Mice: A Role for Remediation of Nitrosative Stress. PLoS One. 2013 Jun 28;8(6):e67358

31. Ailioaie LM, Litscher G, WeberM, Litscher D, Chiran DA. Innovations and Challenges by Applying Sublingual Laser Blood Irradiation in Juvenile Idiopathic Arthritis. Int J of Photoenergy . 2014 may http://dx.doi.org/10.1155/2014/130417

32. Kurtzke, MD. Rating neurologic impairment in multiple sclerosis: An expanded disability status scale (EDSS). NEUROLOGY 1983 Feb 33:1444-52

33. Bryan NS, Grisham MB. Methods to Detect Nitric Oxide and its Metabolites in Biological Samples. Free Radic Biol Med. 2007 Sep 1; 43(5): 645–657.

34. Kevin N. Couper, Daniel G. Blount and Eleanor M. Riley. IL-10: The Master Regulator of Immunity to Infection J Immunol May 1, 2008, 180 (9) 5771-5777; DOI: https://doi.org/10.4049/jimmunol.180.9.5771

35. Rojas JM, Avia M, MartínV, Sevilla N. IL-10: A Multifunctional Cytokine in Viral Infections. J of Immunol Res. 2017 http://dx.doi.org/10.1155/2017/6104054

36. Junior BLM , Delascio RL, Seelaender MCL, Lopes AC. Anti-inflammatory Effect of Physical Training in Heart Failure: Role of TNF-α and IL-10. Arq Bras Cardiol 2009 Out 93(6) : 692-700

37. Macedo RS, Leal MP, Braga TT. Photobiomodulation Therapy Decreases Oxidative Stress in the Lung Tissue after Formaldehyde Exposure: Role of Oxidant/Antioxidant Enzymes. Mediators of Inflammation. 2016 May; http://dx.doi.org/10.1155/2016/9303126.

ANEXO I – Termo de Consentimento Livre e Esclarecido

TCLE - Termo de Consentimento para Participação em Pesquisa Clínica:

Nome do Voluntário:____
Endereço:____
Telefone para contato:__________________Cidade:________________CEP:________
E-mail: ____

1.Título do Trabalho Experimental: A efetividade da fotobiomodulação em indivíduos com Esclerose Múltipla após aplicação na medula espinal e sublingual – Ensaio clínico, controlado e aleatorizado.

2.Objetivo: Avaliar a eficácia da aplicação do laser em baixa intensidade em baixo da língua e na medula espinal em indivíduos com Esclerose Múltipla. Avaliar se a aplicação do laser poderá alterar os níveis óxido nítrico, TNF alfa (substancias inflamatórias) IL-10(Substância anti-inflamatória) e a escala EDSS.

3.Justificativa: Este estudo justifica-se uma vez que se verifica poucos estudos referente ao tratamento com o laser em baixa intensidade em indivíduos com esclerose múltipla.

4. Procedimentos da Fase Experimental: Os participantes serão avaliados por meio da Escala de Status de Expansão Expandida (EDSS) que será aplicada em 15 minutos, e através das amostras de 10 ml de sangue será realizado avaliação da IL-10 (anti-inflamatório), TNF alfa e o óxido nítrico (inflamatório), a coleta de sangue será um procedimento rápido de até 5 minutos. Estas avaliações serão realizadas pré e após tratamento com o laser em baixo da língua e medula espinal. Após as avaliações os participantes serão separados em 4 grupos sendo que o grupo 1 receberá um tratamento fictício com laser em baixo da língua, grupo 2 receberá tratamento com laser em baixo da lingua, grupo 3 o participante receberá tratamento fictício do laser na região da medula, o grupo 4 receberá tratamento com laser na região da medula. É importante ressaltar que os participantes de todos os grupos devem continuar fazendo o tratamento medicamentoso. Para a aplicação do laser em baixo da língua, será utilizado um material plástico descartável cobrindo a caneta de aplicação, por questões de higiene. O tratamento, será realizado 3 vezes por semana totalizando 5 semanas consecutivas. Todos os participantes serão sorteados para fazer parte do grupo que receberá o tratamento real e o grupo que receberá o tratamento fictício. Se houver melhora nos grupos de tratamento com o laser, todos os participantes que forem sorteados para ficarem no grupo do tratamento fictício, receberão o tratamento real com a fotobiomodulação imediatamente após o término do estudo, para que não fique em desvantagem em relação ao outro grupo. Após o tratamento haverá acompanhado por 4 meses, faremos ligações para saber como você está.

5.Desconforto ou Riscos Esperados: Os riscos esperados são mínimos, pois o participante será avaliado e receberá o tratamento em um local fechado, com a presença somente de um familiar (se necessário) e do pesquisador para evitar qualquer tipo de constrangimento. O pesquisador acompanhará o participante em todo trajeto a fim de evitar possíveis quedas. Explicará detalhadamente cada avaliação em que o indivíduo participará e o tratamento que receberá, posicionando-o com cuidado. O pesquisador permanecerá durante toda a avaliação e a aplicação do laser com luvas descartáveis. A pessoa que coletará o seu sangue é habilitada a utilizar os procedimentos adequados para não haver riscos para o(a) sr(a). Entretanto, observamos que há a possibilidade de ocorrer riscos e desconfortos relacionados à coleta venosa, ainda que raros e passageiros, como dor localizada. Raramente desmaio ou infecções no local de punção podem ocorrer. Cuidados devem ser tomados para minimizar esses riscos.

6. Retirada do Consentimento: Retirada do Consentimento: o voluntário tem a liberdade de retirar seu consentimento a qualquer momento e deixar de participar do estudo.

7. Informações: O participante tem a garantia que receberá respostas a qualquer pergunta ou esclarecimento de quaisquer dúvidas quanto aos procedimentos, riscos, benefícios e outros assuntos relacionados com a pesquisa. Também os pesquisadores citados assumem o

compromisso de proporcionar informação atualizada obtida durante o estudo, ainda que esta possa afetar a vontade do indivíduo em continuar participando.

8. Garantia do Sigilo: Os pesquisadores asseguram a privacidade dos participantes quanto aos dados confidenciais envolvidos na pesquisa.

9. Formas de Ressarcimento das Despesas decorrentes da Participação na Pesquisa: Não serão ressarcidas despesas com eventuais deslocamentos.

10. Local da Pesquisa: A pesquisa será desenvolvida nas Clínicas de Fisioterapia da UNINOVE, localizadas nos Campus do Memorial (Rua Dr. Adolfo Pinto, 109 – Barra Funda), Campus da Vila Maria (Rua Profa. Maria José Barone Fernandes, 300 – Vila Maria) e no campus Vergueiro (Rua Vergueiro, 235/249 – Liberdade), São Paulo - SP, Brasil.

11. Comitê de Ética em Pesquisa (CEP) é um colegiado interdisciplinar e independente, que deve existir nas instituições que realizam pesquisas envolvendo seres humanos no Brasil, criado para defender os interesses dos participantes de pesquisas em sua integridade e dignidade e para contribuir no desenvolvimento das pesquisas dentro dos padrões éticos (Normas e Diretrizes Regulamentadoras da Pesquisa envolvendo Seres Humanos – Res. CNS nº 466/12). O Comitê de Ética é responsável pela avaliação e acompanhamento dos protocolos de pesquisa no que corresponde aos aspectos éticos.
Endereço do Comitê de Ética da Uninove: Rua. Vergueiro nº 235/249 – 12º andar - Liberdade – São Paulo – SP CEP. 01504-001 Fone: 3385-9197
comitedeetica@uninove.br

12. Nome Completo e telefones dos Pesquisadores (Orientador e Alunos) para Contato: : Profa. Dra. Sandra Kalil Bussadori (11) 98381-7453 ou Tamiris da Silva (11) 98737-6103.

13. Eventuais intercorrências que vierem a surgir no decorrer da pesquisa poderão ser discutidas pelos meios próprios.

São Paulo, de de .

15. Consentimento Pós-Informação:

Eu, ____, após leitura e compreensão deste termo de informação e consentimento, entendo que minha participação é voluntária, e que posso sair a qualquer momento do estudo, sem prejuízo algum. Confirmo que recebi uma via deste termo de consentimento, e autorizo a realização do trabalho de pesquisa e a divulgação dos dados obtidos somente neste estudo no meio científico.

Nome (por extenso):____

____
Assinatura do Participante / Responsável

16. Eu, ____ (Pesquisador do responsável desta pesquisa), certifico que:

a) Considerando que a ética em pesquisa implica o respeito pela dignidade humana e a proteção devida aos participantes das pesquisas científicas envolvendo seres humanos;
b) Este estudo tem mérito científico e a equipe de profissionais devidamente citados neste termo é treinada, capacitada e competente para executar os procedimentos descritos neste termo;
c) A resolução CNS nº 466/12 dispõe sobre as normas aplicáveis a pesquisas em Ciências Humanas e Sociais, cujo procedimentos metodológicos envolvam a utilização de dados diretamente obtidos com os participantes.

____
Tamiris da Silva
Assinatura do Pesquisador Responsável

1ª via: Instituição

2ª via: Voluntário

ANEXO 2

Escala de Incapacidade Funcional Expandida (EDSS)

Sistemas Funcionais (SF) para a EDSS

Funções Piramidais

0. Normal

1. Sinais anormais sem incapacidade motora

2. Incapacidade mínima

3. Discreta ou moderada paraparesia ou hemiparesia; monoparesia grave

4. Paraparesia ou hemiparesia acentuada; quadriparesia moderada; ou monoplegia

5. Paraplegia, hemiplegia ou acentuada quadriparesia

6. Quadriplegia

V. Desconhecido

Funções Cerebelares

0. Normal

1. Sinais anormais sem incapacidade

2. Ataxia discreta em qualquer membro

3. Ataxia moderada do tronco ou de membros

4. Incapaz de realizar movimentos coordenados devido á ataxia

V. Desconhecido

Funções do Tronco Cerebral

0. Normal

1. Somente sinais anormais

2. Nistagmo moderado ou outra incapacidade leve

3. Nistagmo grave, acentuada paresia extraocular ou incapacidade moderada de outros cranianos

4. Disartria acentuada ou outra incapacidade acentuada

5. Incapacidade de deglutir ou falar

V. Desconhecido

Funções Sensitivas

0. Normal

1. Diminuição de sensibilidade ou estereognosia em 1-2 membros

2. Diminuição discreta de tato ou dor, ou da sensibilidade posicional, e/ou diminuição moderada da vibratória ou estereognosia em 1-2 membros; ou diminuição somente da vibratória em 3-4membros

3. Diminuição moderada de tato ou dor, ou posicional, e/ou perda da vibratória em 1-2 membros; ou diminuição discreta de tato ou dor, e/ou diminuição moderada de toda propriocepção em 3-4 membros

4. Diminuição acentuada de tato ou dor, ou perda da propriocepção em 1-2 membros, ou diminuição moderada de tato ou dor e/ou diminuição acentuada da propriocepção em mais de 2 membros

5. Perda da sensibilidade de 1-2 membros; ou moderada da diminuição de tato ou dor e/ou perda da propriocepção na maior parte do corpo abaixo da cabeça

V. Desconhecido

Funções Vesicais

0. Normal

1. Sintomas urinários sem incontinência

2. Incontinência {ou igual uma vez por semana

3. Incontinência }ou igual uma vez por semana

4. Incontinência diária ou mais que 1 vez por dia

5. Caracterização contínua

6. Grau para bexiga e grau 5 para disfunção retal

V. Desconhecido

Funções intestinais

0. Normal

1. < obstipação diária e sem incontinência

2. Obstipação diária sem incontinência

3. Obstipação < uma vez por semana

4. Incontinência > uma vez por semana mas não diária

5. Sem controle de esfíncter retal

6. Grau 5 para bexiga e grau 5 para disfunção retal

V. desconhecido

Funções Visuais

0. Normal

1. Escotoma com acuidade visual (AV) igual ou melhor que 20/30

2. Pior olho com escotoma e AV de 20/30 a 20/59

3. Pior olho com grande escotoma, ou diminuição moderada dos campos, mas com AV de 20/60 a 20/99

4. Pior olho com diminuição acentuada dos campos e AV de 20/100 a 20/200; ou grau 3 com AV do melhor olho igual ao menor que 20/60

5. Pior olho com AV menor que 20/200; ou grau 4 com AV do melhor olho igual ao menor que 20/60

6. Grau 5 com AV do melhor olho igual ou menor que 20/60

V. Desconhecido

Funções mentais

0. Normal

1. Alterações apenas do humor

2. Diminuição discreta da mentação

3. Diminuição normal da mentação

4. Diminuição acentuada da mentação (moderada síndrome cerebral crônica)

5. Demência ou grave síndrome cerebral crônica

V. Desconhecido

Outras funções

0. Nenhuma

1. Qualquer outro achado devido à EM

2. Desconhecido

Escores EDSS

Escores	Características	Escore Total
0	Exame neurológico normal (todos os SF grau 0; cerebral grau 1 aceitável)	
1,0	Sem incapacidade (1 SF grau 1)	
1,5	Sem incapacidade (2 SF grau 1)	
2,0	Incapacidade mínima em 1 SF (1 SF grau 2, outros grau 0 ou 1)	
2,5	Incapacidade mínima em 2 SF (2 SF grau 2, outros grau 0 ou 1)	

3,0	Incapacidade moderada em 1 SF (1 SF grau 3, outros grau 0 ou 1) ou incapacidade discreta em 3 ou 4 SF (3/4 SF grau 2, outros grau 0 ou 1). Deambulando plenamente.	
3,5	Deambulação plena, com incapacidade moderada em 1SF (1 SF grau 3) e 1 ou 2 SF grau 2; ou 2SF grau 3; ou 5 SF grau 2 (outros 0 ou 1)	
4,0	Deambulação plena, até 500 m sem ajuda ou descanso (1 SF grau 4, outros 0 ou 1)	
4,5	Deambulação plena, até 300 m sem ajuda ou descanso. Com alguma limitação da atividade ou requer assistência mínima (1 SF grau 4, outros 0 ou 1)	
5,0	Deambulação até 200 m sem ajuda ou descanso. Limitação nas atividades diárias (equivalentes são 1 SF grau 5, outros 0 ou 1; ou combinação de graus menores excedendo o escore 4.0)	
5,5	Deambulação até 100 m sem ajuda ou descanso. Incapacidade impedindo atividades plenas diárias (equivalentes são 1SF grau 5, outros 0 ou 1; ou combinações de graus menores excedendo o escore 4.0)	
6,0	Assistência intermitente ou com auxilio unilateral constante de bengala, muleta ou suporte (equivalentes são mais que 2 SF graus 3+)	
6,5	Assistência bilateral (equivalentes são mais que 2 SF graus 3+)	
7,0	Não anda 5 m mesmo com ajuda. Restrito a cadeira de rodas. Transfere da cadeira para cama (equivalentes são combinações com mais que 1 SF 4+, ou piramidal grau 5 isoladamente)	
7,5	Consegue apenas dar poucos passos. Restrito á cadeira de rodas. Necessita ajuda para transferir-se (equivalentes são combinações com mais que 1 SF grau 4+)	
8,0	Restrito ao leito, mas pode ficar fora da cama. Retém funções de autocuidado; bom uso dos braços (equivalentes são combinações de vários SF grau 4+)	
8,5	Restrito ao leito constantemente. Retém algumas funções de autocuidado e dos braços (equivalentes são combinações de vários SF grau 4+)	
9,0	Paciente incapacitado no leito. Pode comunicar, não come, não deglute (equivalentes é a maioria de SF grau 4+)	
9,5	Paciente totalmente incapacitado no leito. Não comunica, não come, não deglute (equivalentes são quase todos de SF grau 4+)	
10	Morte por esclerose múltipla	
**Total**		
