# Supplementary material for: Effects of photobiomodulation on interleukin-10 and nitrites in individuals with relapsing-remitting multiple sclerosis – Randomized clinical trial
Source: PLoS One. 2020 Apr 7;15(4):e0230551. doi: 10.1371/journal.pone.0230551 (PMC7138327; doi:10.1371/journal.pone.0230551)
Supplement: S10 File — (PDF) [file pone.0230551.s010.pdf]

1. **Sample Collection and Handling**

Use a 10 mL vacuette tube (red cap), and 25x8 vacutainer needle (Green) to collect 10 mL of peripheral blood. Centrifuge for 10 minutes in a refrigerated centrifuge at 1,000 X g. Remove serum layer and separate 300 µl into 1.5 mL microtubes and store serum samples at <-80 ° C.

After all collections are performed (pre and post treatment), begin preparing the assay.

1. One day before running ELISA, dilute capture antibody in coating buffer. Add 100 µL of this capture antibody solution to all wells of a 96-well plate, and incubate overnight at 2 ° C to 8 ° C.

2. On the day of the assay, bring all reagents to room temperature (RT) prior to use.

3. Wash plate 4 times with at least 300 µL wash buffer per well and remove residual buffer by firmly tapping plate with absorbent paper down onto absorbent paper. All subsequent washings should be performed similarly.

4. To block non-specific binding and reduce background, add 200 µL Assay Diluent per well.

5. Incubate at RA for 1 hour with shaking in a plate placement device (eg 500 rpm with a circular orbit of 0.3 cm). Any subsequent incubation with shaking should be performed similarly.

6. While the plate is being blocked, prepare standard dilutions and serum sample.

7. Wash plate 4 times with 300 µL Wash Buffer.

8. Add 100 µL / well of standard dilutions to the appropriate wells. add 100 µl / well of serum samples.

9. incubate at RA for 2 hours with shaking.

10. Wash plate 4 times with 300 µL Wash Buffer.

11. Add 100 µL of diluted detection antibody solution to each well, seal plate and incubate at room temperature for 1 hour with shaking.

12. Wash plate 4 times with wash buffer.

13. Add 100 µL of diluted Avidin-HRP solution to each well and incubate at RA for 30 minutes with shaking.

14. Wash plate 5 times with 300 µL Wash Buffer. For this final wash, remove wells in wash buffer for 30 seconds to 1 minute for each wash. This will help to minimize the background.

15. Add 100 µL TMB Substrate Solution and incubate in the dark for 30 minutes or until desired color develops *. Positive wells should turn blue.

16. Stop the reaction by adding 100 µL of Stop Solution to each well. Positive wells should change from blue to yellow.

17. Read absorbance at 450 nm at 15 minutes.
