# Supplementary material for: Effects of photobiomodulation on interleukin-10 and nitrites in individuals with relapsing-remitting multiple sclerosis – Randomized clinical trial
Source: PLoS One. 2020 Apr 7;15(4):e0230551. doi: 10.1371/journal.pone.0230551 (PMC7138327; doi:10.1371/journal.pone.0230551)
Supplement: S11 File — (PDF) [file pone.0230551.s011.pdf]

Protocol

Griess Test

Griess Reagent (Preparation in the dark)

0,1g N-(1-Naphthyl)Ethyl-Enediamine.

1g sulfanilamide

90mL deionized water

2,5mL orthophosphoric acid

Store at 2ºC and 8ºC.

Prepare standard sodium nitrite solution for standard curve:

Dissolve 0,06899g $NaNO_2$ (10mM Sodium Nitrite / 10,000µM) in 100 mL distilled water and place in dark flask at room temperature

1. Perform the 96-well plate assay;
2. Room Temperature Reagents;
3. Prepare the standard curve by the serial dilution form;
4. Use 7 microtubes identified with the different concentrations prepared from the initial nitrite concentration.:

Adicione no primeiro microtubo **8 µL de nitrite (10mM/10.000µM) + 492 µL PBS**

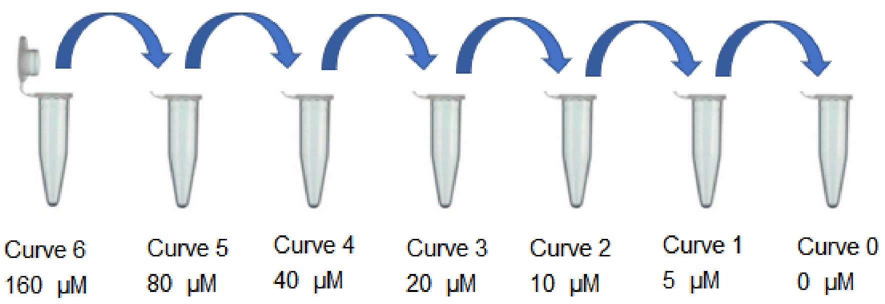

5. Transfer 250 µL from the initial tube to the second to last tube, which must contain 250 µL for serial dilution, since the final volume will be 250 (50 µL for each quadruplicate well);
6. Use the last well to place the blank (supplemented culture medium only);
7. add 50 µL serum sample;
8. add 50µL of Griess reagent to all wells containing samples and standard curve,
9. Wait 10 minutes;
10. Read on 540 nm microplate reader.
